# Does Age Modify the Relation Between Genetic Predisposition to Glaucoma and Various Glaucoma Traits in the UK Biobank?

**DOI:** 10.1167/iovs.66.2.57

**Published:** 2025-02-21

**Authors:** Jihye Kim, Jae H. Kang, Janey L. Wiggs, Hetince Zhao, Keva Li, Nazlee Zebardast, Ayellet Segrè, David S. Friedman, Ron Do, Anthony P. Khawaja, Hugues Aschard, Louis R. Pasquale

**Affiliations:** 1 Department of Epidemiology, Harvard T. H. Chan School of Public Health, Boston, Massachusetts, United States; 2 Channing Division of Network Medicine, Brigham and Women's Hospital/Harvard Medical School, Boston, Massachusetts, United States; 3 Department of Ophthalmology, Harvard Medical School, Massachusetts Eye and Ear Infirmary, Boston, Massachusetts, United States; 4 Department of Ophthalmology, Icahn School of Medicine, Mount Sinai, New York, New York, United States; 5 The Charles Bronfman Institute for Personalized Medicine, Icahn School of Medicine at Mount Sinai, New York, New York, United States; 6 Department of Genetics and Genomic Sciences, Icahn School of Medicine at Mount Sinai, New York, New York, United States; 7 NIHR Biomedical Research Centre at Moorfields Eye Hospital and UCL Institute of Ophthalmology, London, England, United Kingdom; 8 Institut Pasteur, Université de Paris, Department of Computational Biology, Paris, France

**Keywords:** open-angle glaucoma, screening, genetic diseases, intraocular pressure (IOP), nerve fiber layer

## Abstract

**Purpose:**

Glaucoma polygenic risk scores could guide glaucoma public health screening initiatives. We investigated how age influences the relationship between a multitrait glaucoma polygenic risk score (mtGPRS) and primary open-angle glaucoma indicators, including intraocular pressure (IOP), retinal structure, and glaucoma prevalence.

**Methods:**

We analyzed UK Biobank participants with demographic and genetic data, assessing IOP (*n* = 118,153), macular retinal nerve fiber layer thickness (mRNFL; *n* = 42,132), macular ganglion cell inner plexiform layer thickness (mGCIPL; *n* = 42,042), and prevalent glaucoma status (8982 cases among 192,283 participants). An mtGPRS was constructed using 2673 genetic variants. We used multivariable linear regression to assess how age modifies the relationship between mtGPRS and glaucoma traits (IOP, mRFNL, and mGCIPL) and multivariable logistic regression for prevalent glaucoma risk. We analyzed age quartiles (Q1 = <51, Q2 = 51–57, Q3 = 58–62, and Q4 = ≥63 years) – glaucoma trait interaction tests with the Wald test. All analyses were adjusted for confounders, including nonlinear age effects.

**Results:**

Age significantly modified the relationship between the mtGPRS and IOP (*P*_interaction_ = 2.7e-27). Mean IOP differences (millimeters of mercury [mm Hg]) per standard deviation (SD) of mtGPRS were 0.95, 1.02, 1.18, and 1.24 across age quartiles. Similar trends were observed for glaucoma risk (odds ratio per SD of mtGPRS = 2.38, 2.57, 2.80, and 2.75; *P*_interaction_ = 1.0e-06). Relationships between mtGPRS and inner retinal thickness (mRNFL and mGCIPL) across age strata were inconsistently modified by age (*P*_interaction_ ≥ 0.01).

**Conclusions:**

With increasing age, an mtGPRS was a better predictor of higher IOP and glaucoma prevalence. It is useful to consider chronological age with genetic information in designing glaucoma screening strategies.

Primary open-angle glaucoma (POAG) is a complex, intraocular pressure (IOP)-related optic nerve disease that is the most common cause of irreversible blindness worldwide.^1^ Hundreds of genetic variants are associated with the development of POAG.^2^ Although the actual causal genes tagged by these loci remain to be determined, their collective effect, aggregated into a polygenic risk score (PRS), has shown potential utility for diagnosing POAG in various settings.^3^^–^^6^ Furthermore, higher glaucoma PRS is associated with earlier age in treatment,^7^ more visual field progression,^8^ and invasive glaucoma surgery intervention at a younger age.^9^

The main reason why people go blind from glaucoma is because the disease has an insidious onset, and patients present too late.^10^^,^^11^ Genetic makeup is largely determined at birth. Thus, a properly timed recall-by-glaucoma PRS approach might represent a viable screening strategy to facilitate early disease detection. The timing of such a prevention strategy must account for the age-related component of the disease and its fairly low incidence. The 5-year incidence of “at least possible POAG” increased from 0.5% in participants aged 40 to 49 years to 4.1% in participants aged 60 to 69 years among White participants living in Melbourne, Australia.^12^ The 4-year incidence of open-angle glaucoma increased from 1.2% in participants aged 40 to 49 years to 4.2% in participants 70 years old or older among Black people living in Barbados.^13^

PRSs have been developed for other common complex diseases, including colorectal cancer,^14^ cardiovascular disease,^15^ and breast cancer.^16^ For colorectal cancer and cardiovascular disease, a higher PRS produces a stronger odds ratio for disease among younger patients,^14^^,^^15^ whereas for breast cancer overall, age did not seem to modify the relation between the PRS and disease.^16^ Whether age modifies the relation between a PRS and the risk of glaucoma is unknown. Determining the age groups where a PRS most effectively predicts glaucoma risk can help inform cost-effective methods for disease detection. In this study, we examined whether the relation between a multitrait glaucoma polygenic risk score (mtGPRS) and glaucoma, as well as glaucoma-related traits, was modified by age and sex among UK Biobank participants.

## Methods

### Ethics Statement

The UK Biobank received ethical approval from the NHS North West Multicentre Research Ethics Committee (reference number 06/MRE08/65) and the National Information Governance Board for Health and Social Care. This research was conducted under UK Biobank application number 36741 and adhered to the tenets of the Declaration of Helsinki.

### Study Population

We used data from the UK Biobank, a population-based cohort study collected from multiple assessment centers across the United Kingdom. The study includes over 500,000 participants aged 37 to 73 years when recruited through the National Health Service (NHS) registers at baseline (2006–2010). Study participants completed touchscreen questionnaires that provided extensive phenotype information, such as demographic, lifestyle, and medical history information. In addition, participants provided blood, urine, and saliva specimens.

### Genotype Data

Two genotyping platforms were used to generate high throughput allele data on approximately 490,000 individuals. The DNA from most participants (approximately 450,000) was processed on the Affymetrix UK Biobank Axiom Array, which generated genotypes at 825,927 loci, wheres the remaining samples were run with the Affymetrix UK BiLEVE Axiom Array, which provided a similar number of genotypes (807,411).^17^ These platforms were similar and therefore underwent joint quality controls and imputation.^18^ The 1000 Genomes Project, the UK 10 K, and the Haplotype Reference Panel were used to impute genetic architecture. The final dataset contained 92,693,895 genotypes for 487,442 participants.

### Principal Component Analysis-Based ancestry

We conducted a principal component analysis (PCA) on the genotype matrix using the approach proposed by Privé to determine genetically inferred ancestry and generated nine subcontinental groups (UK European, Italy, Poland, Ashkenazi Jewish, India, China, Iran, Nigeria, and the Caribbean).^19^ For our analyses, we grouped those into five ancestry groups: UK European, other European (Italy, Poland, and Ashkenazi Jewish), Asian (India and China), African (Nigeria), and other (Iran and Caribbean). For some additional analyses, we also used the genetically inferred European ancestry variable available in the UK Biobank database (“Genetic ethnic grouping”: Field ID = 22006).

### Genetic Scores of Glaucoma-Related Traits

For each individual, we built a PRS involving 2673 uncorrelated glaucoma-related common single nucleotide polymorphisms (SNPs) after linkage disequilibrium (LD) clumping at *r*^2^ = 0.1 and *P* value ≤ 0.001 identified by a recent multitrait genomewide association study (GWAS) that incorporates variants associated with IOP, cup-disc ratio, and glaucoma status.^3^ The rationale for choosing these SNPs is they provided the highest area under the receiver operator curve for detecting glaucoma in an Australian cohort.^3^ We refer to this as an mtGPRS. We obtained the GWAS summary statistics from the multitrait analysis of glaucoma features^3^ and calculated an mtGPRS with the formula, mtGPRS_(j)_ = ∑jβjG ji  where β_j_ is the log odds ratio of independent trait-related SNP j and G_ji_ is a continuous dosage datum for the risk allele of the SNP j in individual i. For simplicity of interpretation, we normalized it with a mean of 0 and standard deviation (SD) of 1.

In sensitivity analyses, we also tested for interactions between age and trait-specific PRS for four glaucoma traits (IOP, macula region retinal nerve fiber layer [mRNFL] thickness, macula region ganglion cell (mGCIPL) thickness, and glaucoma) with the same formula (i.e. PRS = ∑jβjG ji ) for the mtGPRS but using the genomewide significant SNPs (*P* < 5.0e-08) that were associated with each phenotype. The PRS for IOP, mRNFL, mGCIPL, and glaucoma incorporated 111, 32, 23, and 123 independent common SNPs that had genomewide significant associations with the phenotypes, respectively (see the Supplementary SNP lists). We chose polygenic risk scoring based on SNP panels as opposed to methods that do not rely on *P* value thresholds because we considered several glaucoma traits and not a refined disease phenotype like POAG. Each set of GWAS summary statistics was available from previous GWAS analyses of the individual phenotypes.^20^^–^^22^ As we did for the mtGPRS, each trait-specific PRS was also normalized with a mean of zero and SD of one.

### Glaucoma-Related Outcomes

From 2009 to 2013, ophthalmic data were collected on approximately 115,000 participants in the UK Biobank. The Ocular Response Analyzer (Reichert Corp.) obtained random point estimates of corneal-compensated IOP. Quality control measures instituted to process tonometry data excluded participants with recent ocular infections and a history of glaucoma laser or surgery procedures. We imputed an untreated IOP for participants using ocular hypotensive medicines by multiplying the measured IOP by 1.3, an approach used in prior studies.^22^^,^^23^ Data from the right and left eyes were averaged if measurements from both eyes were available.

At the same time, approximately 65,000 participants submitted to optical coherence tomography (OCT) for inner retinal thickness measurements relevant to glaucoma. Spontaneous mydriasis induced in a dark room facilitated examinations with the Topcon 3D OCT1000 Mark II that captured 3-dimensional 6 × 6 mm^2^ macula volume scans. Macula morphology was reconstructed with 512 A-scans per B scan and 128 horizontal B scans in a grid format. These scans were annotated to yield mRNFL and ganglion cell inner plexiform layer (mGCIPL) thicknesses. Quality control measures undertaken to process these data have been previously described.^24^ As with the IOP data, a mean of right and left eye values was calculated when data from both eyes were available.

### Determination of Prevalent Glaucoma

During the study baseline, participants completed a touchscreen survey displaying the question, “Has a doctor told you that you have any of the following problems with your eyes?” They were considered cases if they chose glaucoma from the menu choices. They were also considered to have glaucoma if they offered a history of glaucoma laser or surgery, or their hospital-linked record indicated a diagnosis code for open-angle glaucoma (International Classification of Disease 9th revision [ICD-9] = 365.* or ICD 10th revision [ICD-10] = H40**, excluding H40.0* and H42*).

### Statistical Analysis

We tabulated characteristics of the study population for the outcome of IOP by quartiles of age (Q1 = <51, Q2 = 51–57, Q3 = 58–62, and Q4 = ≥63 years) using mean and SD for continuous variables and frequency and percentage (%) for categorical variables. Study population characteristics for the other outcomes (glaucoma, mRNFL, and mGCIPL) were also calculated. To explore whether PCA-based ancestry groups in our data might also modify the age-mtGPRS interaction, we checked age-stratified IOP distributions, age distributions, prevalent glaucoma risk, mRNFL, and mGCIPL in each ancestry group.

We assessed age-stratified associations between the mtGPRS and four glaucoma-related outcomes (IOP, mRNFL, mGCIPL, and prevalent glaucoma) using multiple regression models (linear models for IOP, mRNFL, and mGCIPL; logistic models for prevalent glaucoma) adjusting for potential confounding factors. The covariates included age, age^2^, sex, PCA-based ancestry (UK-European, other European, Asian, African, and other), Townsend deprivation index (continuous; higher index score indicates more material deprivation), smoking status (never, past, and current smoker), number of cigarettes smoked per day (continuous; only among current smokers), alcohol drinking frequency (never or special occasion only, 1–3 times per month, 1–2 times per week, and daily or almost daily), coffee and tea intake (cups per day), physical activity (metabolic equivalent of tasks in hours per week), body mass index (BMI; kg/m^2^), systolic blood pressure (millimeters of mercury [mm Hg]), diabetes (yes or no), cardiovascular disease (yes or no), spherical equivalent (continuous in diopters), and beta-blocker use (yes or no). Aside from the main effect of age, the model also included the effect of age^2^, to account for possible nonlinear age effects which could impact the validity of gene-by-age interaction tests. As we also observed that the age-specific associations of mtGPRS and the outcomes depended on the level of the Townsend deprivation index, we added an age-deprivation interaction term in the statistical models.

The significance of both marginal and interaction effect estimates was assessed using Wald tests. Because age appeared to modify the relationships between mtGPRS and glaucoma linearly, we did not model age^2^ – mtGPRS interactions.

We conducted multiple secondary analyses to further characterize the observed interaction effects. First, data for the interaction between age and mtGPRS in the multivariable-adjusted models were calculated for Europeans only, the predominant group in the UK Biobank. Second, comparable age-stratified associations between trait-specific PRSs and glaucoma outcomes (IOP, mRNFL, mGCIPL, and glaucoma) were assessed with similar multivariable regression models. Analyses using trait-specific PRSs involving European-only participants were also performed.

We conducted a sensitivity analysis to see how the interaction effects varied by genetically inferred ancestry (i.e. all UK Biobank participants, European participants only, and non-European participants). For comparison, we repeated the same analysis for IOP. As patients with diabetes mellitus (DM) have higher IOP and increased glaucoma risk and are encouraged to get frequent eye examinations, we also performed additional sensitivity analyses by excluding any glaucoma cases at baseline or excluding participants with DM.

To investigate whether individual biologically important genetic variants of IOP and glaucoma have age-specific effects, we evaluated age-stratified associations between IOP SNPs (rs74315329 on *MYOC*; and rs116089225 and rs10918274 on *TMCO1*) and IOP and between glaucoma SNPs (rs10965235 on *CDKN28-AS1* and rs33912345 on *SIX6*) and prevalent glaucoma. We also assessed an interaction effect of age and each IOP- or glaucoma-related SNP in the association with IOP and glaucoma, respectively.

To examine whether sex modifies the age-specific associations, we performed stratified analyses by sex for age-specific relationships between mtGPRS and the four outcomes and assessed a three-way interaction term of age, sex, and mtGPRS with the Wald test for each outcome. Similarly, we performed age-specific associations between trait-specific PRSs and the four outcomes by sex and evaluated the interaction effects of age, sex, and trait-specific PRSs.

We assessed age-specific associations of mtGPRS and four outcomes in stratified analysis by ancestry groups. To examine whether the age-specific associations are different by ancestry, we included a three-way interaction term of age, mtGPRS, and ancestry to the regression models, and tested it with the Wald test.

All the analyses were performed using the R software and the nominal significance of statistical tests was determined by *P* value < 0.05.

## Results

### Characteristics of UKB Study Participants

For 118,153 UK Biobank study participants with data on IOP, the mean age (SD) was 56.8 (8.0) years. The majority of the participants (≥79%) were UK Europeans across all age groups (Table 1). Compared with younger participants, the oldest participants smoked less, and were more physically active; however, they were more likely to have diabetes and cardiovascular diseases and were more likely to use beta-blocker medicines. Mean IOP (SD) overall was 16.0 (3.8) mm Hg and increased from 15.2 (3.5) mm Hg in participants in the lowest age quartile (<51 years) to 16.7 (4.0) mm Hg in the highest age quartile (≥63 years). Glaucoma prevalence overall was 4.7% and increased from 1.7% in participants in the lowest age quartile (<51 years) to 7.8% in the highest age quartile (≥63 years; Supplementary Table S1). Age-specific characteristics for mRNFL and mGCIPL thicknesses are described in Supplementary Tables S2 and S3. Overall, mean mRNFL (SD) and mean mGCIPL (SD) thicknesses in these populations were 28.9 microns (3.8), and 75.2 microns (5.2), respectively. The distribution of age, glaucoma status, IOP, mRNFL, and mGCIPL as a function of genetically inferred ancestry is shown in Supplementary Figure S1. Participants of African descent were younger than other ancestral groups represented in the UK Biobank but had a higher prevalence of glaucoma and higher IOP in every age category (see Supplementary Fig. S1).

**Table 1.** Age-Stratified Characteristics of the UK Biobank Study Participants With Intraocular Pressure Measurements

	Age^*^			
	<51 Y	51–57 Y	58–62 Y	≥63 Y
**Characteristic**	(*N* = 29,354)	(*N* = 26,271)	(*N* = 27,392)	(*N* = 35,136)
Age, y, mean (SD)	45.5 (3.0)	54.1 (2.0)	60.2 (1.4)	65.7 (2.0)
Female sex, *n* (%)	16,049 (54.7)	14,787 (56.3)	14,820 (54.1)	17,523 (49.9)
Ancestry,^†^ *n* (%)				
UK EUR	23,189 (79.0)	22,500 (85.6)	24,738 (90.3)	32,332 (92.0)
Other EUR	1,225 (4.2)	933 (3.6)	871 (3.2)	869 (2.5)
Asian	1,319 (4.5)	843 (3.2)	563 (2.1)	596 (1.7)
African	908 (3.1)	487 (1.9)	226 (0.8)	264 (0.8)
(Other)	2,713 (9.2)	1,508 (5.7)	994 (3.6)	1,075 (3.1)
Townsend Deprivation Index, mean (SD)	−0.5 (3.1)	−1.0 (3.0)	−1.4 (2.9)	−1.4 (2.8)
Smoking status, *n* (%)				
Never	18,190 (62.0)	15,502 (59.0)	14,865 (54.3)	17,595 (50.1)
Past	7,286 (24.8)	8,075 (30.7)	10,244 (37.4)	15,100 (43.0)
Current	3,878 (13.2)	2,694 (10.3)	2,283 (8.3)	2,441 (6.9)
Alcohol drinking frequency, *n* (%)				
Never or special occasion only	6,388 (21.8)	5,279 (20.1)	5,157 (18.8)	7,561 (21.5)
Ever and often	22,966 (78.2)	20,992 (79.9)	22,235 (81.2)	27,575 (78.5)
Coffee, cups per day, mean (SD)	1.8 (1.8)	1.9 (1.8)	1.9 (1.7)	1.9 (1.7)
Tea, cups per day, mean (SD)	2.9 (2.1)	3.2 (2.1)	3.2 (2.0)	3.2 (2.0)
Physical activity, MET-hours/week, mean (SD)	43.7 (45.1)	42.1 (44.1)	43.4 (43.4)	46.3 (44.2)
Body Mass Index, kg/m^2^, mean (SD)	27.0 (4.6)	27.3 (4.6)	27.4 (4.4)	27.5 (4.3)
Systolic blood pressure, mm Hg, mean (SD)	129.0 (15.7)	135.0 (17.0)	139.8 (17.9)	144.2 (18.3)
Diabetes, *n* (%)	994 (3.4)	1,403 (5.3)	1,778 (6.5)	2,895 (8.2)
Cardiovascular disease, *n* (%)	382 (1.3)	847 (3.2)	1,495 (5.5)	3,084 (8.8)
Systemic beta-blocker use, *n* (%)	581 (2.0)	1,099 (4.2)	1,950 (7.1)	3,591 (10.2)
Spherical equivalent, diopters, mean (SD)	−0.9 (2.5)	−0.6 (2.7)	−0.2 (2.7)	0.3 (2.6)
IOP, mm Hg, mean (SD)^‡^	15.2 (3.5)	15.7 (3.6)	16.3 (3.8)	16.7 (4.0)
Glaucoma at baseline, *n* (%)	178 (0.6)	335 (1.3)	585 (2.1)	1,207 (3.4)
mtGPRS, mean (SD)^§^	0.0 (1.0)	0.0 (1.0)	0.0 (1.0)	0.0 (1.0)

EUR, European; IOP, intraocular pressure; MET, metabolic equivalent task; mtGPRS, multitrait glaucoma polygenic risk score; SD, standard deviation; UK, United Kingdom.

* Age was categorized with quartiles in all UK Biobank amongst participants with available IOP measurements.

† Genetic Principal component analysis based.

‡ IOP represents corneal-compensated values measured with the Ocular Response Analyzer.

§ The mtGPRS polygenic was normalized so that the mean was 0 and the SD was 1.

### Age-Stratified Associations of Multitrait Glaucoma Polygenic Risk Scores and Four Outcomes

**Figure 1.**
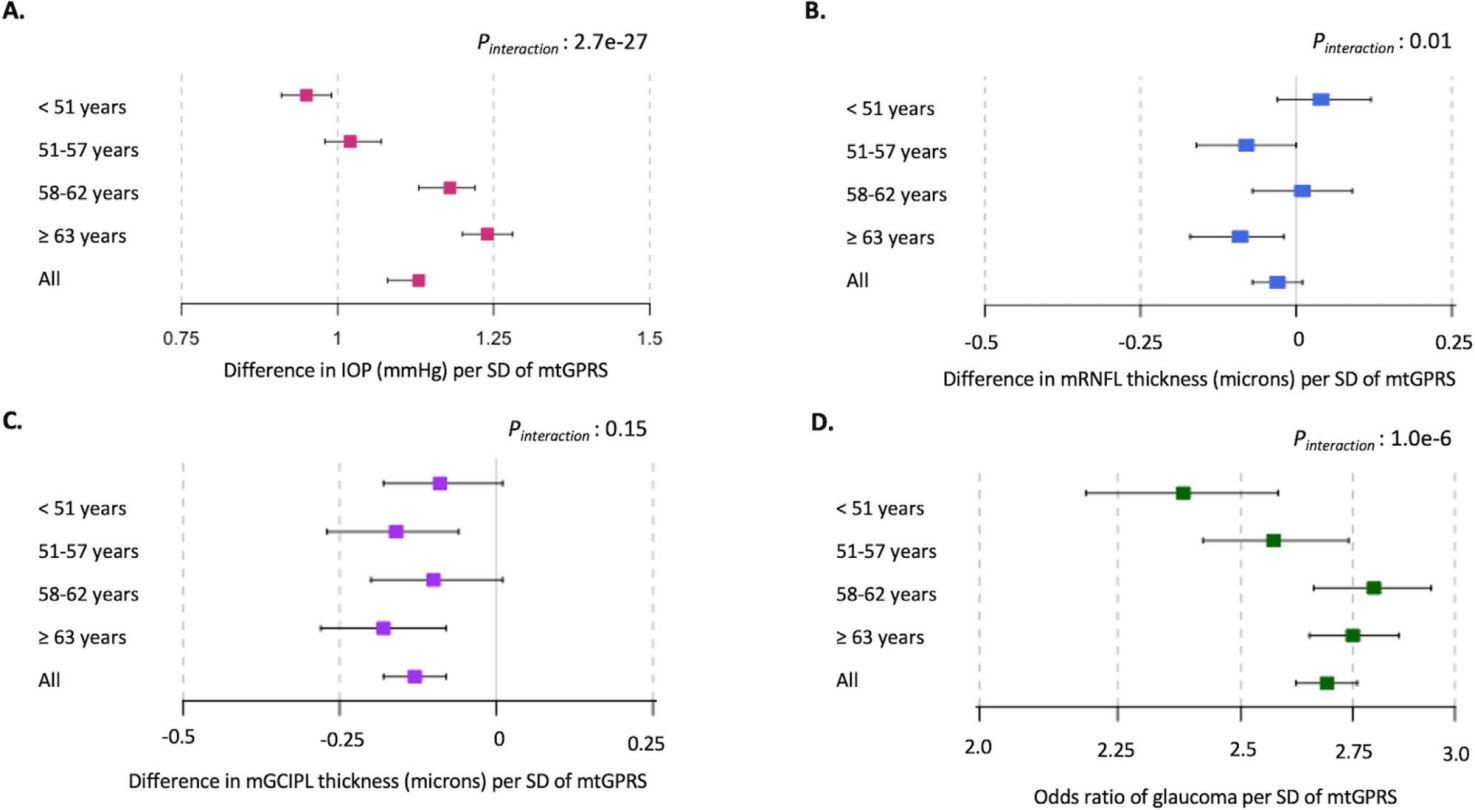
**Age-stratified associations between multitrait glaucoma polygenic risk score (mtGPRS) per standard deviation (SD) and four outcomes in UK Biobank participants.** (**A**) IOP, *n* = 118,153; (**B**) mRNFL thickness, *n* = 42,132; (**C**) mGCIPL thickness, *n* = 42,042; (**D**) glaucoma risk, *n* = 192,283. IOP, intraocular pressure; mGCIPL, macula region ganglion cell complex inner plexiform layer; mtGPRS, multitrait glaucoma polygenic risk score; mRNFL, macula region retinal nerve fiber layer thickness; UK, United Kingdom. Adjusted for age, age^2^, sex, principal component analysis-based ancestry, smoking status (never, past, current), number of cigarettes (only among current smokers), alcohol consumption (never/special occasion only or ever/frequently), physical activity (MET-hours/week), Townsend Deprivation Index, body mass index (kg/m^2^), systolic blood pressure (mm Hg), diabetes mellitus, cardiovascular disease, spherical equivalents (diopters), coffee and tea intake, beta-blocker use, and age* deprivation interaction. NB: *P* value of the interaction test (Wald test) the interaction between mtGPRS and continuous age.

We evaluated associations between the mtGPRS and four outcomes (IOP, mRNFL, mGCIPL, and prevalent glaucoma) by age quartiles, as well as interaction effects of the mtGPRS and continuous age on the outcomes (Fig. 1, Supplementary Table S4). Overall, because we found little confounding by covariates, we solely refer to multivariable-adjusted results in the text. The mtGPRS was strongly related to IOP, with stronger associations for older individuals (<51 years = 0.95 mm Hg vs. ≥63 years: and 1.24 mm Hg per SD increase in mtGPRS). The interaction test between the mtGPRS and continuous age for IOP was highly significant (*P*_interaction_ = 2.7e-27). Similarly, we observed that the mtGPRS was strongly associated with prevalent glaucoma, and the associations were generally stronger with older age (OR per SD increase in mtGPRS: 2.38 to 2.75 for <51 years and ≥63 years, respectively). For glaucoma, we also detected a significant interaction between mtGPRS and age (*P*_interaction_ = 1.0e-6). There were inverse relations between mtGPRS and inner retinal biomarkers (−0.09 microns and –0.18 microns per SD of mtGPRS for mRNFL and mGCIPL, respectively) for participants age ≥63 years (see Supplementary Table S4). There was very limited evidence of mtGPRS-by-age interaction on inner retinal biomarkers (mRNFL *P*_interaction_ = 0.01 and mGCIPL *P*_interaction_ = 0.15). Because age modified the relationship between mtGPRS and glaucoma indicators other than IOP at a much lower significance level, we also explored trait-specific PRSs as the exposure of interest. Age modifications of the relations between glaucoma trait-specific PRSs and our glaucoma outcomes were materially similar to those reported for the mtGPRS in the UK Biobank cohort (Supplementary Fig. S2, Supplementary Table S5).

### Sensitivity Analyses for Age-Stratified Associations of mtGPRS and the Four Outcomes: Europeans, Exclusion of Glaucoma Cases, and Exclusion of Participants With Diabetes Mellitus

**Figure 2.**
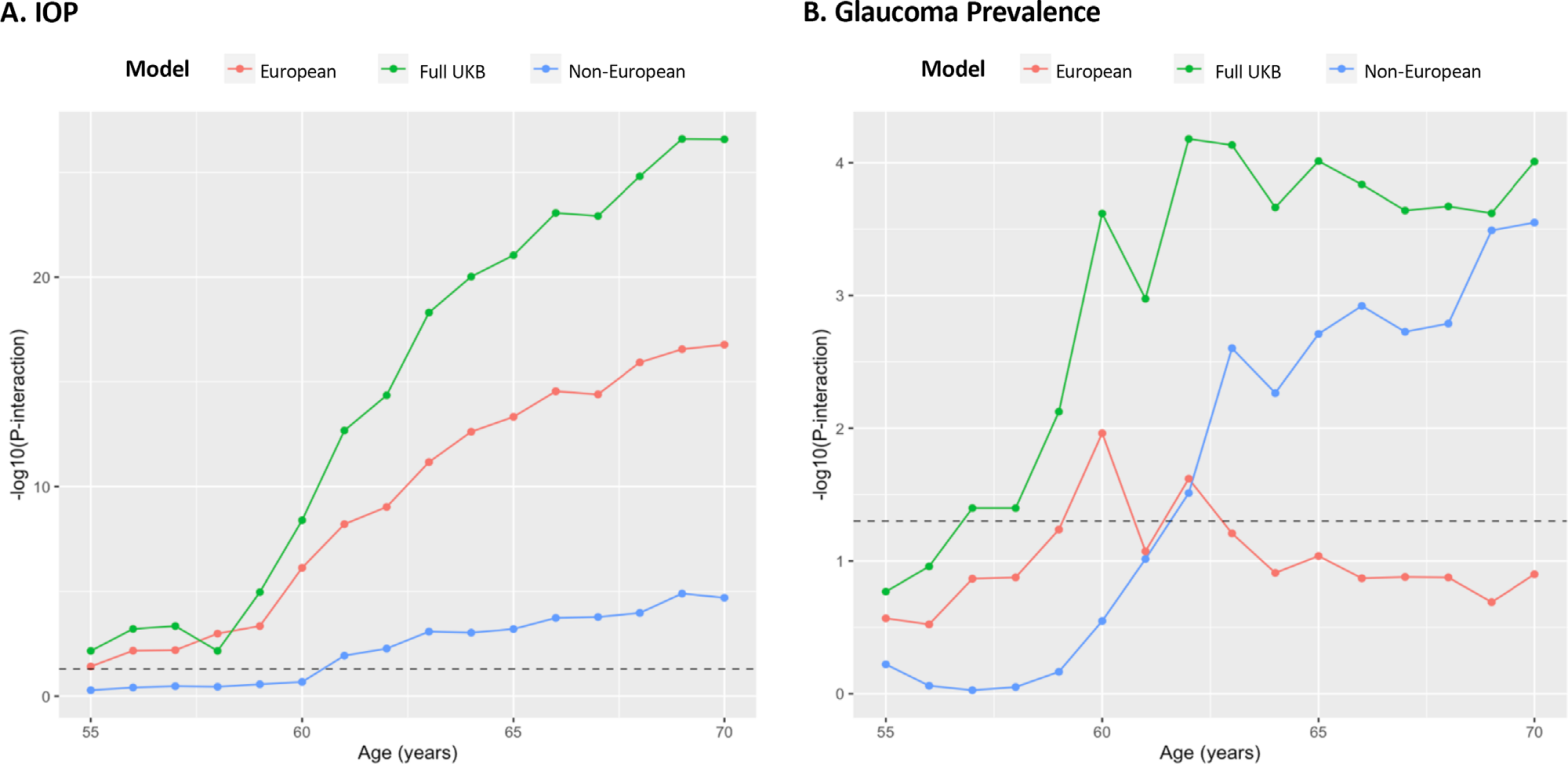
**Sensitivity analysis of interaction effects between multitrait glaucoma polygenic risk score as a function of age**. Europeans (*red*); *N* = 92,693 for IOP, *N* = 154,406 for glaucoma. Non-European UK Biobank participants (*blue*); *N* = 25,460 for IOP, *N* = 37,877 for glaucoma. For (**A**), the *P*-interaction represents the significance of continuous age modifying the relation between mtGPRS and IOP. For (**B**), the *P*-interaction represents the significance of continuous age modifying the relation between mtGPRS and glaucoma. Adjusted for age, age^2^, sex, principal component analysis-based ancestry (this variable was excluded in the subgroup analysis for Europeans and non-Europeans, smoking status (never, past, or current) number of cigarettes (only among current smokers), alcohol consumption (never/special occasion only or ever/frequently), physical activity (MET-hours/week), Townsend Deprivation Index, body mass index (kg/m^2^), systolic blood pressure (mm Hg), diabetes mellitus, cardiovascular disease, spherical equivalents (diopters), coffee and tea intake, beta-blocker use, and age* deprivation. NB: In this analysis, age is adjusted for participants up to age 55 years and then incrementally for each additional chronological year. The *dashed horizontal line* indicates a *P* value of 0.05.

The relation between mtGPRS and IOP as a function of age among Europeans (*P*_interaction_ = 1.7e-17; *N* = 92,693; Supplementary Table S6) was similar compared to the UKBB cohort overall (*P*_interaction_ = 2.7e-27; *N* = 118,153; see Supplementary Table S4). Conversely, the relation between the mtGPRS and glaucoma conditional on age was substantially weaker (*P*_interaction_ = 0.13; 7298 cases among 154,406 participants; see Supplementary Table S6). The results were not importantly altered when the exposure was trait-specific PRSs, as opposed to the mtGPRS (Supplementary Table S7). When illustrated graphically, it is evident that Europeans were strong drivers of the interaction between mtGPRS and continuous age for IOP (Fig. 2A). In contrast, non-European participants were strong drivers for the continuous age – mtGPRS interaction for glaucoma (*P*_interaction_ = 3.2e-04; *N* = 37,877; Fig. 2B).

Age significantly modified an inverse relation between mtGPRS and mRNFL among Europeans (*P*_interaction_ = 8.0e-03; see Supplementary Table S6) but did not modify the relationship between mtGPRS and mGCIPL in the same racial group (*P*_interaction_ = 0.18; see Supplementary Table S6). These trends were not qualitatively altered when trait-specific PRSs, as opposed to the mtGPRS, were the exposures of interest (see Supplementary Table S7).

IOP is a strong genetic endophenotype of glaucoma,^25^ so we explored whether the relationship between mtGPRS and IOP varied as a function of age after excluding participants with glaucoma (*N* = 116,287; Supplementary Table S8). Whether we considered genetically inferred Europeans only or included all ancestral groups, the positive relation between mtGPRS and IOP increased with age (*P*_interaction_ = 9.3e-07; see Supplementary Table S8). The age modification of the inverse relation between mtGPRS and mRNFL (*P*_interaction_ = 0.05; see Supplementary Table S8; *N* = 41,601) persisted after excluding glaucoma cases. Finally, the absence of an effect of age modification on the relation between mtGPRS and mGCIPL was not changed after excluding glaucoma cases.

Finally, patients with DM have higher IOP and increased glaucoma risk,^26^ so we further explored whether age modified the relationship between multitrait GPRS and glaucoma features like IOP (*N* = 111,083) and glaucoma (*N* = 179,232) after excluding participants with DM. Again, we found similar age modifications in these relationships that mirrored the trends we found in the cohort overall (Supplementary Table S9).

### Age-Stratified Associations of Selected Candidate Loci and the Four Glaucoma-Related Outcomes

*MYOC* harbors rare but highly penetrant exon 3 variants with known effects on human trabecular meshwork function^27^ and POAG risk at the population-based level.^28^ An *MYOC* exon 3 variant (rs74315329; effect allele: A) was associated with a 1.63 mm Hg (95% confidence interval [CI] = 0.64 to 2.64) and 2.67 mm Hg (95% CI = 1.7 to 3.60) increased IOP in participants <51 years old and ≥63 years old, respectively, resulting in a nominally significant age-gene interaction (*P*_interaction_ = 0.05; Supplementary Table S10). *TMCO1* contains a common locus with a large effect size for IOP^29^ and *TMCO1* variants predicted conversion from ocular hypertension to POAG in the Ocular Hypertension Treatment Study.^30^ The *TMCO1* variant (rs10918274; effect allele: T) was associated with a 0.34 mm Hg (95% CI = 0.25 to 0.44) and 0.46 mm Hg (95% CI = 0.36 to 0.56) increased IOP in participants <51 years old and ≥63 years old, respectively, resulting in a significant age-gene interaction (*P*_interaction_ = 0.01; see Supplementary Table S10). Conversely, we did not find evidence for interaction between age and *MYOC* or *TMCO1* variants on glaucoma (*P*_interactio_ ≥ 0.09). Two top common variants in *CDKN2B-AS1* and *SIX6*, thought to impact optic nerve vulnerability in POAG,^31^^–^^33^ were significantly associated with glaucoma risk in participants ≥63 years, but the risk did not vary significantly with age (*P*_interaction_ ≥ 0.39; see Supplementary Table S10).

### Sex-Stratified, Age-Stratified Associations of Multitrait Glaucoma Polygenic Risk Score and Four Outcomes

After stratification by sex, age continued to modify the association between mtGPRS and IOP as well as the association between mtGPRS and glaucoma (*P*_interaction_ ≤ 0.01; Table 2); no significant three-way interaction among mtGPRS, continuous age, and sex was evident (*P*_3-way interaction_ ≥ 0.68). Among male subjects, there was no relationship between the mtGPRS and mRNFL thickness across all ages (−0.04 microns per SD of mtGPRS, 95% CI = –0.10 to 0.01). For female subjects, there was an inverse association between mtGPRS and mRNFL thickness in participants 63 years and older (−0.14 microns per SD of mtGPRS, 95% CI = −0.25 to −0.03]) yielding both a significant interaction with age (*P*_interaction_ = 0.001) as well as a significant 3-way interaction between mtGPRS, continuous age and sex (*P*_3-way interaction_ = 0.04). In female subjects 63 years of age and older, there was an inverse relationship between mtGPRS and mGCIPL that was stronger with older age (*P*_interaction_ = 0.04); no 3-way interaction among mtGPRS, continuous age, and sex existed (*P*_3-way interaction_ = 0.16). When age-specific associations between trait-specific PRSs and the four glaucoma-related outcomes were stratified by sex, no significant three-way interactions were evident (*P*_3-way interaction_ ≥ 0.22; Supplementary Table S11). When age-specific associations between mtGPRS and the four glaucoma traits were stratified by ancestry, no significant interactions among mtGPRS, age, and ancestry were noted (Supplementary Table S12; *P*_3-way interaction_ ≥ 0.09).

**Table 2.** Age-Stratified Associations Between Multitrait Glaucoma Polygenic Risk Score (mtGPRS) Per Standard Deviation (SD) and Four Outcomes by Sex Among UK Biobank Participants

	Male			Female			
Age^*^	*N*	Beta [95% CI] Per SD of mtGPRS	*P_interaction_* ^†^	N	Beta [95% CI] Per SD of mtGPRS	*P_interaction_* ^†^	*P_interaction_* ^‡^
IOP, mm Hg							
All	54,974	1.17 [1.13 to 1.20]	2.9 e-14	63,179	1.05 [1.02 to 1.08]	3.9 e-13	0.72
<51 y	13,305	1.01 [0.95 to 1.07]		16,049	0.89 [0.84 to 0.95]		
51–57 y	11,484	1.05 [0.98 to 1.12]		14,787	1.00 [0.94 to 1.05]		
58–62 y	12,572	1.24 [1.18 to 1.31]		14,820	1.12 [1.06 to 1.18]		
≥63 y	17,613	1.29 [1.24 to 1.36]		17,523	1.17 [1.11 to 1.23]		
mRNFL thickness (microns)
All	19,939	−0.04 [−0.10 to 0.01]	0.69	22,193	−0.01 [−0.07 to 0.04]	0.001	0.04
<51 y	5,344	0.01 [−0.10 to 0.11]		5,996	0.09 [−0.02 to 0.20]		
51–57 y	4,237	−0.17 [−0.29 to −0.05]		5,270	0.003 [−0.11 to 0.11]		
58–62 y	4,314	0.03 [−0.09 to 0.14]		5,030	0.001 [−0.11 to 0.11]		
≥63 y	6,044	−0.05 [−0.15 to 0.05]		5,897	−0.14 [−0.25 to −0.03]		
mGCIPL thickness (microns)
All	19,868	−0.17 [−0.25 to −0.10]	0.99	22,174	−0.10 [−0.17 to −0.03]	0.04	0.16
<51 y	5,301	−0.15 [−0.29 to −0.01]		6,001	−0.03 [−0.16 to 0.11]		
51–57 y	4,220	−0.26 [−0.42 to −0.10]		5,265	−0.09 [−0.24 to 0.05]		
58–62 y	4,308	−0.12 [−0.27 to 0.04]		5,012	−0.11 [−0.26 to 0.03]		
≥63 y	6,039	−0.18 [−0.32 to −0.05]		5,896	−0.18 [−0.31 to −0.04]		
		**SD of mtGPRS**			**SD of mtGPRS**		
	**N Case/N Total**	**OR [95% CI] Per**	*P_interaction_* ^†^	**N Case/N Total**	**OR [95% CI] Per**	*P_interaction_* ^†^	*P_interaction_* ^‡^

Glaucoma risk							
All	4,680/88,713	2.75 [2.66 to 2.85]	0.01	4,302/103,570	2.62 [2.52 to 2.72]	0.004	0.68
<51 y	422/21,483	2.45 [2.21 to 2.73]		362/25,420	2.28 [2.02 to 2.58]		
51–57 y	706/18,668	2.58 [2.37 to 2.82]		685/23,771	2.57 [2.35 to 2.81]		
58–62 y	1,147/19,995	2.93 [2.73 to 3.14]		1,107/24,222	2.69 [2.51 to 2.90]		
≥63 y	2,405/28,567	2.81 [2.67 to 2.96]		2,148/30,157	2.69 [2.54 to 2.84]		

CI, confidence interval; IOP, intraocular pressure; mRNFL, macula region retinal nerve fiber layer; mGCIPL, macula region ganglion cell inner plexiform layer thickness; OR, odds ratio; y, years.

* Categorization based on the quartiles of age in the full UK Biobank amongst participants with available IOP measurements.

† *P* value of the interaction test (Wald test) between MTAG PRS and continuous age.

‡ *P* value of the three-way interaction (Wald test) of MTAG PRS, continuous age, and sex.

## Discussion

IOP is a polygenic trait strongly related to POAG.^22^ A higher IOP PRS is a risk factor for higher IOP and POAG.^4^ A higher IOP PRS has been associated with more severe glaucoma, the need for more glaucoma surgery, and a larger number of family members affected.^34^ Our work extends those observations by showing that increasing age consistently amplifies the relationship between a mtGPRS and IOP in both sexes. This is remarkable as the multivariable models control for both age and age^2^ to consider possible nonlinear relationships. It is unclear whether age modifies the effect of mtGPRS scores in non-European ancestral groups, as they are under-represented in the UK Biobank. Similar results were obtained after excluding participants with glaucoma or participants with DM, who tend to have higher IOP, are at higher risk of glaucoma, and are recommended to get more frequent eye examinations (see Supplementary Tables S8, S9). When an IOP PRS was substituted for the mtGPRS in sensitivity analysis, we also found similar results (see Supplementary Table S5). A selected single rare variant with the largest known IOP effect size (*MYOC*, rs74315329) and a top common variant with a more modest IOP effect (*TMCO1*, rs10918274) also exhibited stronger effects in older participants that were statistically significant (P_interaction_ ≤ 0.05; see Supplementary Table S10). The consistent increase in effects of the mtGPRS and IOP PRS on IOP as a function of age may partially explain why POAG is a strongly age-related disease.

POAG has emerged as a polygenic disease,^2^^,^^3^ and, as for most common human diseases, aggregating disease-related variants into PRS has become the primary strategy for the translation of genetic findings to public health. Yet, disentangling the relationship between such scores and glaucoma by age, sex, and ancestral groups could help refine glaucoma screening strategies. We find that among participants younger than 51 years old, the mtGPRS increased glaucoma risk by 2.35-fold per each SD whereas it increased glaucoma risk by 2.75-fold per SD for participants 63 years of age and older. This effect of modification by age was statistically significant and it could have implications for enhancing the positive predictive strategy for glaucoma screening. Assuming the population prevalence of POAG increases from 2% to 4% for people <51 years and people >63 years, respectively, then the glaucoma prevalence in these age brackets increases from 9.4% to 22% for people at the highest 2 SD cutoff for mtGPRS. Using a 2-tier approach to screen a population where >1 in 5 have glaucoma might be cost-effective, although cases among those with lower genetic risk will still be missed. After stratification by age, further subdivision by sex did not yield additional insights into glaucoma screening methods.

Interestingly, non-Europeans, not participants of European descent, were the main drivers of the age-mtGPRS interaction on glaucoma (see Fig. 2B). Detection bias may explain this trend as non-Europeans tended to be younger than Europeans among study participants and younger people may be less likely to get screened for glaucoma; furthermore, the prevalence of glaucoma was higher in people of color versus Europeans (see Supplementary Fig. 1B). IOP-independent factors may explain why the mtGPRS increased glaucoma risk with age in non-Europeans as age only weakly modified the relationship between mtGPRS and IOP in non-Europeans. Importantly, the sample size for the non-Europeans was substantially smaller than for the Europeans, so some of the observed differences might be explained by a lack of statistical power and larger datasets will be required to assess these findings.

RNFL and GCIPL thicknesses are inner retinal biomarkers that decline with progressive glaucoma. Mendelian Randomization experiments suggest that genetic determinants of these biomarkers are not in the causal pathway of glaucoma.^20^ As expected, trait-specific PRSs for mRNFL and mGCIPL have strong positive associations with the respective thicknesses of these inner retinal layers but these relationships did not vary noticeably by age (see Fig. 1^2^). A higher mtGPRS was associated with an inverse association with mRNFL thickness only in participants ≥63 years old. We found a more consistent inverse relation between mtGPRS and mGCIPL, but this inverse relation did not become significantly stronger with age. We can infer from these findings that the mtGPRS does not have profound direct associations with the tissues targeted in glaucoma optic neuropathy. Rather, it may be the consequence of a strong genetic predisposition to elevated IOP, which produces loss of inner retinal tissue in glaucoma. Interestingly, a study that integrated gene regulation and single-cell RNA sequence to gain functional insight into glaucoma genomewide significant loci did not nominate retinal ganglion cells or their axons, the tissues that comprise the RNFL and GCIPL.^35^

People <51 years of age had a 0.95 mm Hg higher IOP per SD of mtGPRS and people ≥63 years had a 1.24 mm Hg higher IOP per SD of mtGPRS (*P*_interaction_ = 2.7e-27). This strong modification effect raises the question of whether IOP itself increases as a function of age, regardless of genetic status. Data on the longitudinal change in IOP as a function of age yields mixed results, with reports of mostly IOP decreases^36^^–^^41^ and some increases^42^^,^^43^ with age in individuals of Asian ancestry, whereas others reported increases of IOP with age in individuals of African ancestry^44^ and Europeans.^45^^,^^46^ In this cross-sectional, largely European cohort, IOP did increase with age (see Table 1), and in every age stratum, individuals of African ancestry had higher IOP than people of other ancestries (see Supplementary Fig. S1C). Reportedly, genetic heritability to higher IOP increases with age but the trend was inconsistent.^47^ We estimated IOP SNP heritability (*h^2^*) in unrelated UK Biobank participants of European ancestry using LD score regression (LDSC) but did not observe any significant trend with increasing age (Supplementary Table S13).^48^ This suggests a combination of genetic and non-genetic factors may result in the strong modifying effect of age on genetic predisposition to higher IOP. Exactly how aging impacts the genes involved in determining IOP levels is unknown.

Strengths of this study include the large sample size which allowed us to explore how age, an important risk factor for POAG, modified the relation between mtGPRS and IOP as well as glaucoma. All sensitivity analyses, including the uses of trait-specific PRSs, investigations of interaction effects of age on top IOP loci-IOP relationships, and excluding patients with glaucoma and patients with DM who tend to have higher IOP, yielded consistent results.

This work also has some limitations. First, the UK Biobank displays a slight healthy volunteer bias, which could impact the observed PRS-by-age interaction effects. Second, the limited sample size for non-European ancestries does not allow for detailed ancestry-specific age-mtGPRS interactions, and our results may not be generalizable to non-Europeans. Third, UK Biobank participants range in age from 37 to 73 years. It would be of interest to assess age modification of the genetic risk of glaucoma for participants outside this age range. Fourth, case confirmation of self-reported glaucoma in the UK Biobank is not available, and misclassification of glaucoma undoubtedly occurred, but such misclassification would drive our results to the null, and we find that the definition of glaucoma produces consistent results across the spectrum of age and ancestry (see Supplementary Tables S12 and S14). Although different PRS methods exist that use all variants,^49^ our SNP panel method that relied on *P* value thresholds was more suitable for this analysis and does not change the fundamental conclusion that age modifies the relationship between glaucoma genetic predisposition and key glaucoma features.

Overall, our study demonstrates that genetic predisposition to higher IOP and glaucoma is amplified by older age. As genetic risk scoring for glaucoma becomes more readily available, those with the highest genetic predisposition need to be prioritized for glaucoma screening. In the UK Biobank, those in the 58 to 62-year-old age group had the highest odds of having glaucoma. This information is useful for planning screening strategies for those currently undiagnosed individuals in the most vulnerable populations. Finally, resilience biomarkers related to younger age hold promise for mitigating the genetic predisposition to higher IOP and glaucoma in older individuals.

## Supplementary Material

Supplement 1

## References

[1] Tham YC, Li X, Wong TY, Quigley HA, Aung T, Cheng CY. Global prevalence of glaucoma and projections of glaucoma burden through 2040: a systematic review and meta-analysis. *Ophthalmology*. 2014; 121: 2081–2090. 24974815 10.1016/j.ophtha.2014.05.013

[2] Han X, Gharahkhani P, Hamel AR, et al. Large-scale multitrait genome-wide association analyses identify hundreds of glaucoma risk loci. *Nat Genet*. 2023; 55: 1116–1125. 37386247 10.1038/s41588-023-01428-5 PMC10335935

[3] Craig JE, Han X, Qassim A, et al. Multitrait analysis of glaucoma identifies new risk loci and enables polygenic prediction of disease susceptibility and progression. *Nat Genet*. 2020; 52: 160–166. 31959993 10.1038/s41588-019-0556-y PMC8056672

[4] Gao XR, Huang H, Kim H. Polygenic risk score is associated with intraocular pressure and improves glaucoma prediction in the UK Biobank cohort. *Transl Vis Sci Technol*. 2019; 8: 10. 10.1167/tvst.8.2.10 PMC6450641 30972231

[5] Hsiao YJ, Chuang HK, Chi SC, et al. Genome-wide polygenic risk score for predicting high risk glaucoma individuals of Han Chinese ancestry. *J Pers Med*. 2021; 11: 1169. 34834521 10.3390/jpm11111169 PMC8618593

[6] Liu Q, Davis J, Han X, et al. Cost-effectiveness of polygenic risk profiling for primary open-angle glaucoma in the United Kingdom and Australia. *Eye (Lond)*. 2023; 37: 2335–2343. 36513856 10.1038/s41433-022-02346-2 PMC10366078

[7] Marshall HN, Mullany S, Han X, et al. High polygenic risk is associated with earlier initiation and escalation of treatment in early primary open-angle glaucoma. *Ophthalmology*. 2023; 130: 830–836. 37044160 10.1016/j.ophtha.2023.03.028

[8] Siggs OM, Qassim A, Han X, et al. Association of high polygenic risk with visual field worsening despite treatment in early primary open-angle glaucoma. *JAMA Ophthalmol*. 2022; 141: 73–77. 36355370 10.1001/jamaophthalmol.2022.4688 PMC9650622

[9] Marshall HN, Hollitt GL, Wilckens K, et al. High polygenic risk is associated with earlier trabeculectomy in patients with primary open-angle glaucoma. *Ophthalmol Glaucoma*. 2023; 6: 54–57. 35842105 10.1016/j.ogla.2022.06.009

[10] Grant WM, Burke JF Jr. Why do some people go blind from glaucoma? *Ophthalmology*. 1982; 89: 991–998. 7177577 10.1016/s0161-6420(82)34675-8

[11] Susanna R Jr., De Moraes CG, Cioffi GA, Ritch R. Why do people (still) go blind from glaucoma? *Transl Vis Sci Technol*. 2015; 4: 1. 10.1167/tvst.4.2.1 PMC4354096 25767744

[12] Mukesh BN, McCarty CA, Rait JL, Taylor HR. Five-year incidence of open-angle glaucoma: the visual impairment project. *Ophthalmology*. 2002; 109: 1047–1051. 12045042 10.1016/s0161-6420(02)01040-0

[13] Leske MC, Connell AM, Wu SY, et al. Incidence of open-angle glaucoma: the Barbados Eye Studies. The Barbados Eye Studies Group. *Arch Ophthalmol*. 2001; 119: 89–95. 11146731

[14] Thomas M, Sakoda LC, Hoffmeister M, et al. Genome-wide modeling of polygenic risk score in colorectal cancer risk. *Am J Hum Genet*. 2020; 107: 432–444. 32758450 10.1016/j.ajhg.2020.07.006 PMC7477007

[15] Jiang X, Holmes C, McVean G. The impact of age on genetic risk for common diseases. *PLoS Genet*. 2021; 17: e1009723. 34437535 10.1371/journal.pgen.1009723 PMC8389405

[16] Mavaddat N, Michailidou K, Dennis J, et al. Polygenic risk scores for prediction of breast cancer and breast cancer subtypes. *Am J Hum Genet*. 2019; 104: 21–34. 30554720 10.1016/j.ajhg.2018.11.002 PMC6323553

[17] Wain LV, Shrine N, Miller S, et al. Novel insights into the genetics of smoking behaviour, lung function, and chronic obstructive pulmonary disease (UK BiLEVE): a genetic association study in UK Biobank. *Lancet Respir Med*. 2015; 3: 769–781. 26423011 10.1016/S2213-2600(15)00283-0 PMC4593935

[18] Bycroft C, Freeman C, Petkova D, et al. The UK Biobank resource with deep phenotyping and genomic data. *Nature*. 2018; 562: 203–209. 30305743 10.1038/s41586-018-0579-z PMC6786975

[19] Prive F, Aschard H, Carmi S, et al. Portability of 245 polygenic scores when derived from the UK Biobank and applied to 9 ancestry groups from the same cohort. *Am J Hum Genet*. 2022; 109: 12–23. 34995502 10.1016/j.ajhg.2021.11.008 PMC8764121

[20] Currant H, Hysi P, Fitzgerald TW, et al. Genetic variation affects morphological retinal phenotypes extracted from UK Biobank optical coherence tomography images. *PLoS Genet*. 2021; 17: e1009497. 33979322 10.1371/journal.pgen.1009497 PMC8143408

[21] Gharahkhani P, Jorgenson E, Hysi P, et al. Genome-wide meta-analysis identifies 127 open-angle glaucoma loci with consistent effect across ancestries. *Nat Commun*. 2021; 12: 1258. 33627673 10.1038/s41467-020-20851-4 PMC7904932

[22] Khawaja AP, Cooke Bailey JN, Wareham NJ, et al. Genome-wide analyses identify 68 new loci associated with intraocular pressure and improve risk prediction for primary open-angle glaucoma. *Nat Genet*. 2018; 50: 778–782. 29785010 10.1038/s41588-018-0126-8 PMC5985943

[23] Stuart KV, Luben RN, Warwick AN, et al. The association of alcohol consumption with glaucoma and related traits: findings from the UK Biobank. *Ophthalmol Glaucoma*. 2023; 6: 366–379. 36481453 10.1016/j.ogla.2022.11.008 PMC10239785

[24] Madjedi KM, Stuart KV, Chua SYL, et al. The association of physical activity with glaucoma and related traits in the UK Biobank. *Ophthalmology*. 2023; 130: 1024–1036. 37331483 10.1016/j.ophtha.2023.06.009 PMC10913205

[25] Tran JH, Stuart KV, de Vries V, et al. Genetic associations between smoking- and glaucoma-related traits. *Transl Vis Sci Technol*. 2023; 12: 20. 10.1167/tvst.12.2.20 PMC9932549 36786746

[26] Zhao D, Cho J, Kim MH, Friedman DS, Guallar E. Diabetes, fasting glucose, and the risk of glaucoma: a meta-analysis. *Ophthalmology*. 2015; 122: 72–78. 25283061 10.1016/j.ophtha.2014.07.051

[27] Zode GS, Kuehn MH, Nishimura DY, et al. Reduction of ER stress via a chemical chaperone prevents disease phenotypes in a mouse model of primary open angle glaucoma. *J Clin Invest*. 2011; 121: 3542–3553. 21821918 10.1172/JCI58183 PMC3163970

[28] Zebardast N, Sekimitsu S, Wang J, et al. Characteristics of p.Gln368Ter myocilin variant and influence of polygenic risk on glaucoma penetrance in the UK Biobank. *Ophthalmology*. 2021; 128: 1300–1311. 33713785 10.1016/j.ophtha.2021.03.007 PMC9134646

[29] Van Koolwijk LM, Ramdas WD, Ikram MK, et al. Common genetic determinants of intraocular pressure and primary open-angle glaucoma. *PLoS Genet*. 2012; 8: e1002611. 22570627 10.1371/journal.pgen.1002611 PMC3342933

[30] Scheetz TE, Faga B, Ortega L, et al. Glaucoma risk alleles in the ocular hypertension treatment study. *Ophthalmology*. 2016; 123: 2527–2536. 27707548 10.1016/j.ophtha.2016.08.036 PMC5121091

[31] Burdon KP, Crawford A, Casson RJ, et al. Glaucoma risk alleles at CDKN2B-AS1 are associated with lower intraocular pressure, normal-tension glaucoma, and advanced glaucoma. *Ophthalmology*. 2012; 119: 1539–1545. 22521085 10.1016/j.ophtha.2012.02.004

[32] Carnes MU, Liu YP, Allingham RR, et al. Discovery and functional annotation of SIX6 variants in primary open-angle glaucoma. *PLoS Genet*. 2014; 10: e1004372. 24875647 10.1371/journal.pgen.1004372 PMC4038608

[33] Pasquale LR, Loomis SJ, Kang JH, et al. CDKN2B-AS1 genotype-glaucoma feature correlations in primary open-angle glaucoma patients from the United States. *Am J Ophthalmol*. 2013; 155: 342–353.e345. 23111177 10.1016/j.ajo.2012.07.023 PMC3544983

[34] Qassim A, Souzeau E, Siggs OM, et al. An intraocular pressure polygenic risk score stratifies multiple primary open-angle glaucoma parameters including treatment intensity. *Ophthalmology*. 2020; 127: 901–907. 32081492 10.1016/j.ophtha.2019.12.025

[35] Hamel AR, Yan W, Rouhana JM, et al. Integrating genetic regulation and single-cell expression with GWAS prioritizes causal genes and cell types for glaucoma. *Nat Commun*. 2024; 15: 396. 38195602 10.1038/s41467-023-44380-y PMC10776627

[36] Asaoka R, Obana A, Murata H, et al. The association between age and systemic variables and the longitudinal trend of intraocular pressure in a large-scale health examination cohort. *Invest Ophthalmol Vis Sci*. 2022; 63: 22. 10.1167/iovs.63.11.22 PMC9624273 36301531

[37] Baek SU, Kee C, Suh W. Longitudinal analysis of age-related changes in intraocular pressure in South Korea. *Eye (Lond)*. 2015; 29: 625–629. 25697455 10.1038/eye.2015.11 PMC4429270

[38] Chua J, Chee ML, Chin CWL, et al. Inter-relationship between ageing, body mass index, diabetes, systemic blood pressure and intraocular pressure in Asians: 6-year longitudinal study. *Br J Ophthalmol*. 2019; 103: 196–202. 29632002 10.1136/bjophthalmol-2018-311897 PMC6362803

[39] Han X, Zhao H, Wu C, et al. Ten-year changes of intraocular pressure in adults: the Liwan Eye Study. *Clin Exp Ophthalmol*. 2019; 47: 41–48. 30091181 10.1111/ceo.13372

[40] Nakano T, Tatemichi M, Miura Y, Sugita M, Kitahara K. Long-term physiologic changes of intraocular pressure: a 10-year longitudinal analysis in young and middle-aged Japanese men. *Ophthalmology*. 2005; 112: 609–616. 15808252 10.1016/j.ophtha.2004.10.046

[41] Zhao D, Kim MH, Pastor-Barriuso R, et al. A longitudinal study of age-related changes in intraocular pressure: the Kangbuk Samsung Health Study. *Invest Ophthalmol Vis Sci*. 2014; 55: 6244–6250. 25183763 10.1167/iovs.14-14151

[42] Han X, Yang T, Zhang J, et al. Longitudinal changes in intraocular pressure and association with systemic factors and refractive error: Lingtou Eye Cohort Study. *BMJ Open*. 2018; 8: e019416. 10.1136/bmjopen-2017-019416 PMC5829881 29444785

[43] Wang YX, Xu L, Zhang XH, You QS, Zhao L, Jonas JB. Five-year change in intraocular pressure associated with changes in arterial blood pressure and body mass index. The Beijing Eye Study. *PLoS One*. 2013; 8: e77180. 24146967 10.1371/journal.pone.0077180 PMC3795645

[44] Hennis A, Wu SY, Nemesure B, Leske MC, Barbados Eye Studies Group. Hypertension, diabetes, and longitudinal changes in intraocular pressure. *Ophthalmology*. 2003; 110: 908–914. 12750088 10.1016/S0161-6420(03)00075-7

[45] Astrom S, Stenlund H, Linden C. Intraocular pressure changes over 21 years - a longitudinal age-cohort study in northern Sweden. *Acta Ophthalmol*. 2014; 92: 417–420. 23902137 10.1111/aos.12232

[46] Hartmann A, Scholz I, Hoffmann EM, et al. Change of intraocular pressure over 5 years and its relationship to cardiovascular parameters: results from the Gutenberg Health Study. *Invest Ophthalmol Vis Sci*. 2024; 65: 12. 10.1167/iovs.65.1.12 PMC10774695 38175640

[47] Ge T, Chen CY, Neale BM, Sabuncu MR, Smoller JW. Phenome-wide heritability analysis of the UK Biobank. *PLoS Genet*. 2017; 13: e1006711. 28388634 10.1371/journal.pgen.1006711 PMC5400281

[48] Bulik-Sullivan BK, Loh PR, Finucane HK, et al. LD Score regression distinguishes confounding from polygenicity in genome-wide association studies. *Nat Genet*. 2015; 47: 291–295. 25642630 10.1038/ng.3211 PMC4495769

[49] Sekimitsu S, Wang J, Elze T, Segre AV, Wiggs JL, Zebardast N. Interaction of background genetic risk, psychotropic medications, and primary angle closure glaucoma in the UK Biobank. *PLoS One*. 2022; 17: e0270530. 35763501 10.1371/journal.pone.0270530 PMC9239437

