# Supplementary material for: Does Age Modify the Relation Between Genetic Predisposition to Glaucoma and Various Glaucoma Traits in the UK Biobank?
Source: Invest Ophthalmol Vis Sci. 2025 Feb 21;66(2):57. doi: 10.1167/iovs.66.2.57 (PMC11855177; doi:10.1167/iovs.66.2.57)
Supplement: Supplement 1 [file iovs-66-2-57_s001.pdf]

Figure S1. Distributions by principal component-based ancestry groups.

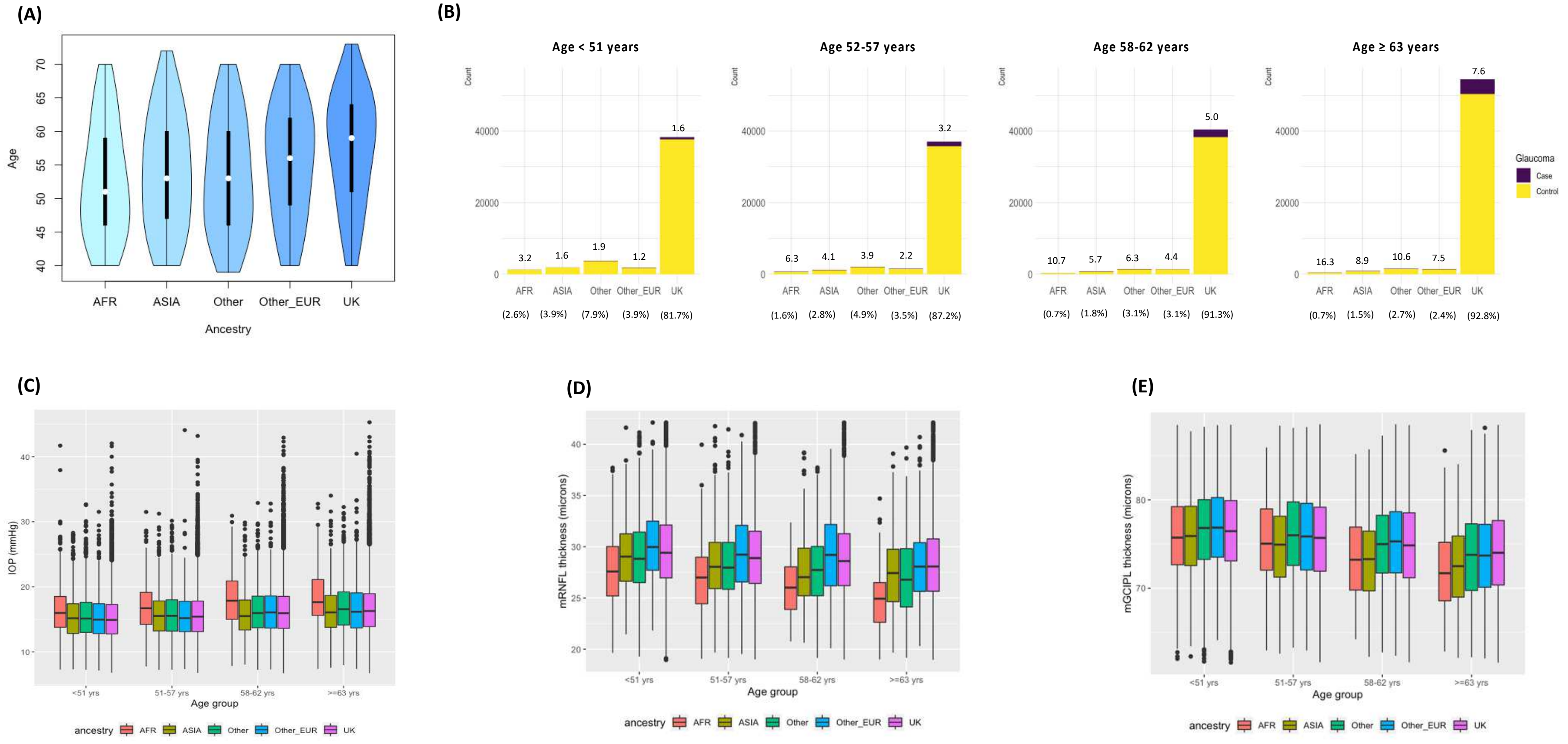

Abbreviations: AFR, African; ASIA, Asian; Other_EUR, other European than UK; UK, United Kingdom

(A) Age: violin plot to show age distributions by ancestry groups in the UK biobank participants with glaucoma data (n=192,283); (B) Glaucoma prevalence: bar charts of glaucoma cases and controls by ancestry groups in each age stratum. The numbers on the top of bars indicate the percentages of cases in each ancestry group and the numbers below represent the percentages of ancestry in each age stratum; (C) Intraocular pressure (IOP): box plot for IOP distribution of each ancestry group by age strata (n=118,153); (D) Macula retinal nerve fiber layer (mRNFL) thickness: box plot for mRNFL distribution of each ancestry group by age strata (n=42,132); (E) Macular ganglion cell inner plexiform layer (mGCIPL) thickness: box plot for mGCIPL distribution of each ancestry group by age strata (n=42,042). Different colors represent different ancestry groups in (C), (D), and (E).

Figure S2. Age-stratified associations between trait-specific polygenic risk scores (PRSs) per standard deviation (SD) and four outcomes among UK Biobank participants.

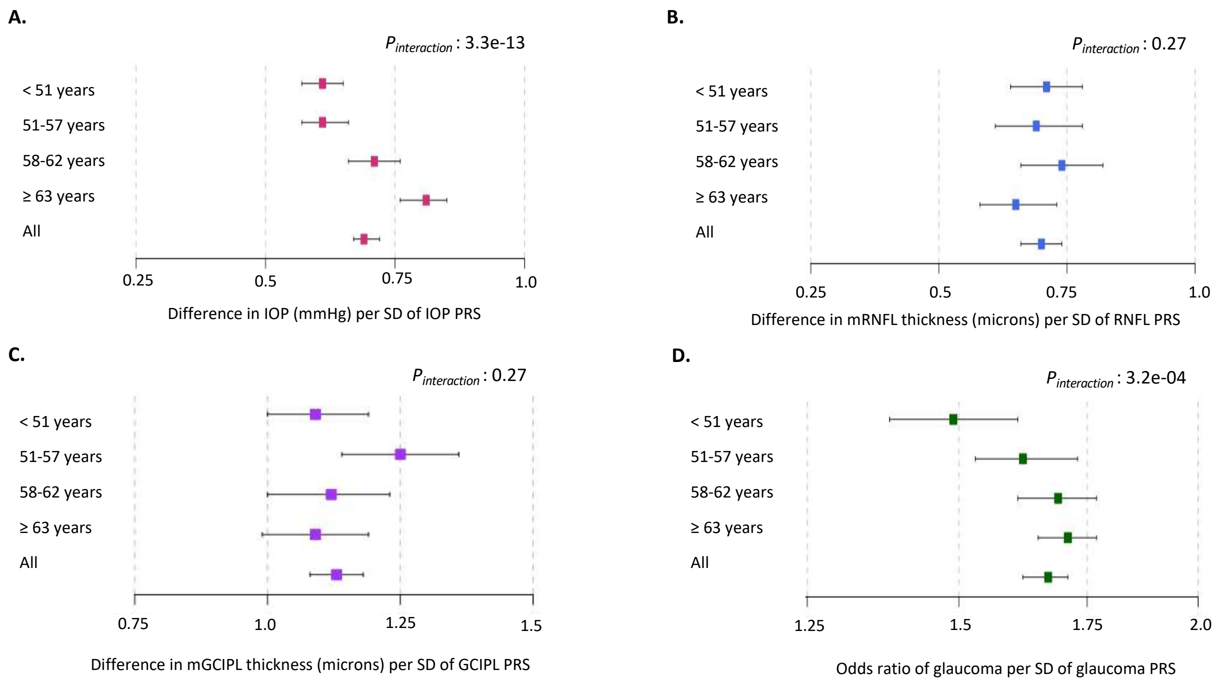

Abbreviations: IOP, intraocular pressure; mRNFL, macula region retinal nerve fiber layer thickness; mGCIPL, macula region ganglion cell inner plexiform thickness.

(A) IOP, n=118,153; (B) mRNFL thickness, n=42,132; (C) mGCIPL thickness, n=42,042; (D) glaucoma risk, n=192,283.

Adjusted for age, age^2, sex, principal component analysis-based ancestry, smoking status (never, past, current), number of cigarettes (only among current smokers), alcohol consumption (never/special occasion only or ever/frequently), physical activity (MET-hours/week), Townsend Deprivation Index, body mass index (kg/m^2), systolic blood pressure (mmHg), diabetes, cardiovascular disease, spherical equivalents (diopters), coffee and tea intake, beta-blocker use, and age*deprivation.

NB: P-value of the interaction test (Wald test) between trait-specific PRS and continuous age. See the supplemental SNP lists for gene variants comprising the trait-specific PRSs.

S1 Table. Age-stratified characteristics of UK Biobank study participants with data on glaucoma status.

	Age[*]			
**Characteristic**	**< 51 years** (N = 46,903)	**51-57 years** (N = 42,439)	**58-62 years** (N = 44,217)	**≥ 63 years** (N = 58,724)
Age (years), mean (SD)	45.6 (3.0)	54.1 (2.0)	60.2 (1.4)	65.8 (2.0)
Female sex, n (%)	25,420 (54.2)	23,771 (56.0)	24,222 (54.8)	30,157 (51.4)
Ancestry[†], n (%)				
UK EUR	38,305 (81.7)	37,008 (87.2)	40,375 (91.3)	54,475 (92.8)
Other EUR	1,806 (3.9)	1,483 (3.5)	1,368 (3.1)	1,400 (2.4)
Asian	1,836 (3.9)	1,195 (2.8)	776 (1.8)	867 (1.5)
African	1,232 (2.6)	668 (1.6)	309 (0.7)	423 (0.7)
Other	3,724 (7.9)	2,085 (4.9)	1,389 (3.1)	1,559 (2.7)
Townsend Deprivation Index, mean (SD)	-0.6 (3.2)	-1.0 (3.1)	-1.4 (3.0)	-1.4 (2.9)
Smoking status, n (%)				
Never	28,862 (61.5)	24,691 (58.2)	23,651 (53.5)	29,358 (50.0)
Past	11,578 (24.7)	13,072 (30.8)	16,681 (37.7)	25,200 (42.9)
Current	6,463 (13.8)	4,676 (11.0)	3,885 (8.8)	4,166 (7.1)
Alcohol drinking frequency, n (%)				
Never or special occasion only	10,126 (21.6)	8,637 (20.4)	8,748 (19.8)	13,394 (22.8)
Ever and often	36,777 (78.4)	33,802 (79.6)	35,469 (80.2)	45,330 (77.2)
Coffee (cups per day), mean (SD)	1.8 (1.8)	1.9 (1.8)	1.9 (1.7)	1.9 (1.7)
Tea (cups per day), mean (SD)	2.9 (2.1)	3.2 (2.1)	3.2 (2.1)	3.2 (2.0)
Physical activity (MET-hours/week), mean (SD)	43.6 (45.7)	41.7 (44.5)	43.1 (43.8)	45.8 (44.3)
Body Mass Index (kg/m^2), mean (SD)	27.0 (4.6)	27.4 (4.7)	27.5 (4.5)	27.6 (4.3)
Systolic blood pressure (mmHg), mean (SD)	129.4 (15.7)	135.3 (17.1)	139.8 (17.8)	144.2 (18.4)
Diabetes, n (%)	1,824 (3.9)	2,614 (6.2)	3,213 (7.3)	5,400 (9.2)
Cardiovascular disease, n (%)	695 (1.5)	1,551 (3.7)	2,675 (6.0)	5,655 (9.6)
Beta-blocker use, n (%)	1,069 (2.3)	2.048 (4.8)	3,423 (7.7)	6,535 (11.1)
Spherical equivalent (diopters), mean (SD)	-0.7 (2.0)	-0.5 (2.2)	-0.2 (2.2)	0.0 (2.1)
IOP (mmHg), mean (SD)[‡]	15.2 (3.5)	15.7 (3.6)	16.3 (3.8)	16.6 (4.0)
Glaucoma at baseline, n (%)	784 (1.7)	1,391 (3.3)	2,254 (2.1)	4,553 (7.8)
mtGPRS, mean (SD)[§]	0.0 (1.0)	0.0 (1.0)	0.0 (1.0)	0.0 (1.0)

Abbreviations: SD, standard deviation; UK, United Kingdom; EUR, European; MET, metabolic equivalent task; IOP, intraocular pressure; mtGPRS, multitrait glaucoma polygenic risk score.

[*] Age was categorized with quartiles in all UK Biobank amongst participants with available IOP measurements.

[†] Principal component analysis based.

[‡] IOP represents corneal-compensated values measured with the Ocular Response Analyzer.

[§] The mtGPRS polygenic was normalized so that the mean was 0 and the SD was 1.

S2 Table. Age-stratified characteristics of UK Biobank study participants with mRNFL measurements.

Characteristic	Age[*]			
	**< 51 years** (N = 11,340)	**51-57 years** (N = 9,507)	**58-62 years** (N = 9,344)	**≥ 63 years** (N = 11,941)
Age (years), mean (SD)	45.4 (3.1)	54.1 (2.0)	60.2 (1.4)	65.6 (2.0)
Female sex, n (%)	5,996 (52.9)	5,270 (55.4)	5,030 (53.8)	5,897 (49.4)
Ancestry[†], n (%)				
UK EUR	9,076 (80.0)	8,215 (86.4)	8,427 (90.2)	11,092 (92.9)
Other EUR	491 (4.3)	331 (3.5)	328 (3.5)	298 (2.5)
Asian	404 (3.6)	237 (2.5)	165 (1.8)	133 (1.1)
African	339 (3.0)	199 (2.1)	74 (0.8)	75 (0.6)
Other	1,030 (9.1)	525 (5.5)	350 (3.7)	343 (2.9)
Townsend Deprivation Index, mean (SD)	-0.5 (3.1)	-1.0 (3.0)	-1.3 (2.8)	-1.4 (2.8)
Smoking status, n (%)				
Never	6,785 (59.8)	5,522 (58.1)	4,961 (53.1)	5,903 (49.4)
Past	3,008 (26.5)	2,998 (31.5)	3,592 (38.4)	5,195 (43.5)
Current	1,547 (13.6)	987 (10.4)	791 (8.5)	843 (7.1)
Alcohol drinking frequency, n (%)				
Never or special occasion only	2,337 (20.6)	1,848 (19.4)	1,726 (18.5)	2,514 (21.1)
Ever and often	9,003 (79.4)	7,659 (80.6)	7,618 (81.5)	9,427 (78.9)
Coffee (cups per day), mean (SD)	1.8 (1.8)	1.9 (1.8)	1.9 (1.7)	1.9 (1.7)
Tea (cups per day), mean (SD)	2.8 (2.1)	3.2 (2.1)	3.2 (2.0)	3.2 (2.0)
Physical activity (MET-hours/week), mean (SD)	45.2 (46.1)	43.2 (45.0)	44.0 (43.9)	47.0 (45.8)
Body Mass Index (kg/m^2), mean (SD)	27.0 (4.6)	27.3 (4.5)	27.4 (4.4)	27.5 (4.2)
Systolic blood pressure (mmHg), mean (SD)	128.8 (15.7)	134.7 (17.1)	139.8 (18.1)	144.3 (18.3)
Diabetes, n (%)	337 (3.0)	428 (4.5)	558 (6.0)	851 (7.1)
Cardiovascular disease, n (%)	139 (1.2)	291 (3.1)	487 (5.2)	980 (8.2)
Beta-blocker use, n (%)	217 (1.9)	381 (4.0)	696 (7.4)	1,206 (10.1)
Spherical equivalent (diopters), mean (SD)	-0.5 (1.7)	-0.2 (1.9)	0.2 (2.0)	0.6 (2.0)
IOP (mmHg), mean (SD)[‡]	15.1 (3.4)	15.6 (3.6)	16.2 (3.6)	16.5 (3.8)
Glaucoma at baseline, n (%)	45 (0.4)	92 (1.0)	135 (1.4)	317 (2.7)
mtGPRS, mean (SD)[§]	0.0 (1.0)	0.0 (1.0)	0.0 (1.0)	0.0 (1.0)

Abbreviations: SD, standard deviation; UK, United Kingdom; EUR, European; MET, metabolic equivalent task; IOP, intraocular pressure; mtGPRS, multitrait glaucoma polygenic risk score.

[*] Age was categorized with quartiles in all UK Biobank amongst participants with available IOP measurements.

[†] Principal component analysis based.

[‡] IOP represents corneal-compensated values measured with the Ocular Response Analyzer.

[§] The mtGPRS polygenic was normalized so that the mean was 0 and the SD was 1.

S3 Table. Age-stratified characteristics of UK Biobank study participants with mGCIPL measurements.

Characteristic	Age[*] < 51 years (N = 11,302)	51-57 years (N = 9,485)	58-62 years (N = 9,320)	≥ 63 years (N = 11,935)
Age (years), mean (SD)	45.4 (3.1)	54.1 (2.0)	60.2 (1.4)	65.6 (2.0)
Female sex, n (%)	6,001 (53.1)	5,265 (55.5)	5,012 (53.8)	5,896 (49.4)
Ancestry[†], n (%)				
UK EUR	9,051 (80.1)	8,192 (86.4)	8,405 (90.2)	11,077 (92.8)
Other EUR	492 (4.4)	332 (3.5)	328 (3.5)	299 (2.5)
Asian	399 (3.5)	235 (2.5)	165 (1.8)	136 (1.1)
African	339 (3.0)	201 (2.1)	72 (0.8)	77 (0.6)
(Other)	1,021 (9.0)	525 (5.5)	350 (3.8)	346 (2.9)
Townsend Deprivation Index, mean (SD)	-0.5 (3.1)	-1.0 (3.0)	-1.3 (2.8)	-1.4 (2.8)
Smoking status, n (%)				
Never	6,776 (60.0)	5,515 (58.1)	4,948 (53.1)	5,901 (49.4)
Past	2,988 (26.4)	2,994 (31.6)	3,585 (38.5)	5,188 (43.5)
Current	1,538 (13.6)	976 (10.3)	787 (8.4)	846 (7.1)
Alcohol drinking frequency, n (%)				
Never or special occasion only	2,330 (20.6)	1,848 (19.5)	1,722 (18.5)	2,512 (21.0)
Ever and often	8,972 (79.4)	7,637 (80.5)	7,598 (81.5)	9,423 (79.0)
Coffee (cups per day), mean (SD)	1.8 (1.8)	1.9 (1.8)	1.9 (1.7)	1.9 (1.7)
Tea (cups per day), mean (SD)	2.8 (2.1)	3.2 (2.1)	3.2 (2.0)	3.2 (2.0)
Physical activity (MET-hours/week), mean (SD)	45.0 (45.8)	43.1 (44.9)	43.9 (43.7)	47.1 (46.0)
Body Mass Index (kg/m^2), mean (SD)	27.0 (4.6)	27.3 (4.5)	27.4 (4.4)	27.5 (4.2)
Systolic blood pressure (mmHg), mean (SD)	128.9 (15.7)	134.7 (17.1)	139.8 (18.1)	144.2 (18.3)
Diabetes, n (%)	332 (2.9)	431 (4.5)	554 (5.9)	853 (7.1)
Cardiovascular disease, n (%)	139 (1.2)	289 (3.0)	487 (5.2)	973 (8.2)
Beta-blocker use, n (%)	215 (1.9)	381 (4.0)	690 (7.4)	1,195 (10.0)
Spherical equivalent (diopters), mean (SD)	-0.5 (1.7)	-0.3 (1.9)	0.1 (2.0)	0.6 (2.0)
IOP (mmHg), mean (SD)[‡]	15.1 (3.4)	15.6 (3.6)	16.2 (3.7)	16.5 (3.8)
Glaucoma at baseline, n (%)	45 (0.4)	91 (1.0)	135 (1.4)	312 (2.6)
mtGPRS, mean (SD)[§]	0.0 (1.0)	0.0 (1.0)	0.0 (1.0)	0.0 (1.0)

Abbreviations: SD, standard deviation; UK, United Kingdom; EUR, European; MET, metabolic equivalent task; IOP, intraocular pressure; mtGPRS, multitrait glaucoma polygenic risk score.

[*] Age was categorized with quartiles in all UK Biobank amongst participants with available IOP measurements.

[†] Principal component analysis based.

[‡] IOP represents corneal-compensated values measured with the Ocular Response Analyzer.

[§] The mtGPRS polygenic was normalized so that the mean was 0 and the SD was 1.

S4 Table[*]. Age-stratified associations between the multitrait glaucoma polygenic risk score (mtGPRS) per standard deviation (SD) and four outcomes with age interactions in UK Biobank participants.

	Unadjusted model			Adjusted model	
	**IOP (mm Hg)**				
**Age[†]**	**N**	**Beta [95% CI] per SD of mtGPRS**	**P-value**	**Beta [95% CI] per SD of mtGPRS**	**P-value**
All	118,153	1.13 [1.11, 1.15]	< 1.0 e-324	1.13 [1.08, 1.13]	< 1.0 e-324
< 51 y	29,354	0.99 [0.95, 1.03]	< 1.0 e-324	0.95 [0.91, 0.99]	< 1.0 e-324
51-57 y	26,271	1.06 [1.02, 1.10]	< 1.0 e-324	1.02 [0.98, 1.07]	< 1.0 e-324
58-62 y	27,392	1.20 [1.16, 1.25]	< 1.0 e-324	1.18 [1.13, 1.22]	< 1.0 e-324
≥ 63 y	35,136	1.28 [1.24, 1.32]	< 1.0 e-324	1.24 [1.20, 1.28]	< 1.0 e-324
$P_{interaction}$[‡]		5.6 e-10		2.7 e-27	
	**mRNFL thickness (microns)**				
All	42,132	-0.02 [-0.05, 0.02]	0.40	-0.03 [-0.07, 0.01]	0.17
< 51 y	11,340	0.01 [-006, 0.08]	0.77	0.04 [-0.03, 0.12]	0.24
51-57 y	9,507	-0.08 [-0.16, -0.01]	0.03	-0.08 [-0.16, 0.00]	0.06
58-62 y	9,344	0.004 [-0.07, 0.08]	0.92	0.01 [-0.07, 0.09]	0.75
≥ 63 y	11,941	-0.05 [-0.12, 0.01]	0.12	-0.09 [-0.17, -0.02]	0.01
$P_{interaction}$[‡]		0.25		0.01	
	**mGCIPL thickness (microns)**				
All	42,042	-0.17 [-0.22, -0.12]	3.7 e-12	-0.13 [-0.18, -0.08]	2.3 e-07
< 51 y	11,302	-0.12 [-0.21, -0.03]	0.01	-0.09 [-0.18, 0.01]	0.08
51-57 y	9,485	-0.22[-0.32, -0.12]	2.7 e-05	-0.16 [-0.27, -0.06]	0.003
58-62 y	9,320	-0.20 [-0.30, -0.10]	0.0001	-0.10 [-0.20, 0.01]	0.06
≥ 63 y	11,935	-0.26 [-0.35, -0.18]	6.9 e-09	-0.18 [-0.28, -0.08]	0.0003
$P_{interaction}$[‡]		0.02		0.15	
	**Glaucoma risk**				
	**N case/ N total**	**OR [95% CI] per SD of mtGPRS**	**P-value**	**OR [95% CI] per SD of mtGPRS**	**P-value**
All	8,982/ 192,283	2.67 [2.61, 2.73]	< 0.001	2.69 [2.62, 2.76]	< 1.0 e-300
< 51 y	784/ 46,903	2.39 [2.22, 2.57]	< 0.001	2.38 [2.19, 2.58]	7.1 e-100
51-57 y	1,391/ 42,439	2.57 [2.43, 2.72]	< 0.001	2.57 [2.42, 2.74]	4.4 e-198
58-62 y	2,254/ 44,217	2.79 [2.67, 2.92]	< 0.001	2.80 [2.66, 2.94]	< 1.0 e-300
≥ 63 y	4,553/ 58,724	2.76 [2.67, 2.85]	< 0.001	2.75 [2.65, 2.86]	< 1.0 e-300
$P_{interaction}$[‡]		1.3 e-04		1.0 e-06	

[*]This data is graphically presented in Fig 1.

Abbreviations: y, years; IOP, intraocular pressure; mRNFL, macula region retinal nerve fiber layer; mGCIPL, macula region ganglion cell inner plexiform layer; OR, odds ratio; CI, confidence interval.

[†] Categorization based on the quartiles of age in the full UK Biobank amongst participants with available IOP measurements.

‡ P-value of the interaction test (Wald test) between mtGPRS and continuous age.
The unadjusted model represents raw computes. The adjusted model controls for age, age^2, sex, principal component analysis-based ancestry, smoking status (never, past, current), number of cigarettes (only among current smokers), alcohol consumption (never/special occasion only or ever/frequently), physical activity(MET-hours/week), Townsend Deprivation Index, body mass index. (kg/m^2), systolic blood pressure (mmHg), diabetes, cardiovascular disease, spherical equivalents (diopters), coffee and tea intake, beta-blocker use, and age*deprivation.

S5 Table*. Age-stratified associations between trait-specific polygenic risk score (PRS) per standard deviation (SD) and four outcomes among UK Biobank participants.

Age†		Unadjusted model		Adjusted model	
		**IOP (mm Hg)**			
	**N**	**Beta [95% CI] per SD of IOP PRS**	**P-value**	**Beta [95% CI] per SD of IOP PRS**	**P-value**
All	118,153	0.71 [0.69, 0.73]	< 1.0 e-324	0.69 [0.67, 0.72]	< 1.0 e-324
< 51 y	29,354	0.63 [0.59, 0.67]	6.2 e-213	0.61 [0.57, 0.65]	4.7 e-177
51-57 y	26,271	0.65 [0.61, 0.70]	4.5 e-191	0.61 [0.57, 0.66]	1.3 e-145
58-62 y	27,392	0.73 [0.69, 0.78]	1.1 e-223	0.71 [0.66, 0.76]	1.6 e-176
≥ 63 y	35,136	0.82 [0.78, 0.86]	< 1.0 e-324	0.81 [0.76, 0.85]	9.1 e-271
$P_{interaction}$‡		3.9 e-13		3.3 e-13	
		**mRNFL thickness (microns)**			
	**N**	**Beta [95% CI] per SD of mRNFL PRS**	**P-value**	**Beta [95% CI] per SD of mRNFL PRS**	**P-value**
All	42,132	0.67 [0.64, 0.71]	7.3 e-290	0.70 [0.66, 0.74]	6.1 e-271
< 51 y	11,340	0.65 [0.59, 0.72]	1.5 e-77	0.71 [0.64, 0.78]	4.6 e-80
51-57 y	9,507	0.65 [0.58, 0.73]	1.5 e-64	0.69 [0.61, 0.78]	8.8 e-62
58-62 y	9,344	0.72 [0.64, 0.79]	3.1 e-75	0.74 [0.66, 0.82]	1.1 e-69
≥ 63 y	11,941	0.65 [0.58, 0.72]	9.6 e-78	0.65 [0.58, 0.73]	1.2 e-65
$P_{interaction}$‡		0.84		0.27	
		**mGCIPL thickness (microns)**			
	**N**	**Beta [95% CI] per SD of mGCIPL PRS**	**P-value**	**Beta [95% CI] per SD of mGCIPL PRS**	**P-value**
All	42,042	1.07 [1.02, 1.12]	< 1.0e-324	1.13 [1.08, 1.18]	< 1.0 e-324
< 51 y	11,302	1.00 [0.91, 1.09]	5.29E-106	1.09 [1.00, 1.19]	3.8 e-109
51-57 y	9,485	1.14 [1.04, 1.25]	4.70E-103	1.25 [1.14, 1.36]	7.1 e-109
58-62 y	9,320	1.07 [0.97, 1.17]	1.67E-89	1.12 [1.00, 1.23]	1.6 e-84
≥ 63 y	11,935	1.03 [0.94, 1.12]	4.79E-107	1.09 [0.99, 1.19]	5.9 e-102
$P_{interaction}$‡		0.80		0.27	
		**Glaucoma risk**			
	**N case/ N total**	**OR [95% CI] per SD of glaucoma PRS**	**P-value**	**OR [95% CI] per SD of glaucoma PRS**	**P-value**
All	8,982/ 192,283	1.54 [1.51, 1.57]	< 0.001	1.67 [1.62, 1.71]	< 1.0 e-300
< 51 y	784/ 46,903	1.45 [1.37, 1.54]	< 0.001	1.49 [1.38, 1.61]	4.6 e-23
51-57 y	1,391/42,439	1.54 [1.47, 1.62]	< 0.001	1.62 [1.53, 1.73]	2.1 e-54
58-62 y	2,254/ 44,217	1.63 [1.57, 1.70]	< 0.001	1.69 [1.61, 1.77]	1.1 e-99
≥ 63 y	4,553/ 58,724	1.66 [1.61, 1.71]	< 0.001	1.71 [1.65, 1.77]	3.6 e-188
$P_{interaction}$‡		8.1 e-05		3.2 e-04	

* This data is graphically presented in S2 Fig.

Abbreviations: PRS, polygenic risk score; IOP, intraocular pressure; y, year; mRNFL, macula retinal nerve fiber layer; mGCIPL, macula region ganglion cell inner plexiform layer; OR, odds ratio; CI, confidence interval.

† Categorization based on the quartiles of age in the full UK Biobank amongst participants with available IOP measurements.

‡ P-value of the interaction test (Wald test) between the trait-specific PRS and continuous age.

The unadjusted model represents raw computes. The adjusted model controls for age, age^2, sex, principal component analysis-based ancestry, smoking status (never, past, current), number of cigarettes (only among current smokers), alcohol consumption (never/special occasion only or ever/frequently), physical activity (MET-hours/week), Townsend Deprivation Index, body mass index. (kg/m^2), systolic blood pressure (mmHg), diabetes, cardiovascular disease, spherical equivalents (diopters), coffee and tea intake, beta-blocker use, and age*deprivation. See the supplemental SNP lists for gene variants comprising the trait-specific PRSs.

S6 Table. Age-stratified associations between mtGPRS per standard deviation (SD) for each glaucoma outcome with interaction analysis among genetically inferred Europeans in the UK Biobank.

Age[*]	Unadjusted model			Adjusted model	
	**IOP (mm Hg)**				
	**N**	**Beta [95% CI] per SD of GPRS**	**P-value**	**Beta [95% CI] per SD of GPRS**	**P-value**
All	92,693	1.20 [1.18, 1.22]	< 1.0 e-324	1.16 [1.14, 1.19]	< 1.0 e-324
< 51 y	20,300	1.07 [1.03, 1.11]	< 1.0 e-324	1.02 [0.97, 1.07]	< 1.0 e-324
51-57 y	21,197	1.12 [1.07, 1.17]	< 1.0 e-324	1.08 [1.03, 1.13]	< 1.0 e-324
58-62 y	22,549	1.25 [1.20, 1.29]	< 1.0 e-324	1.22 [1.18, 1.27]	< 1.0 e-324
≥ 63 y	29,647	1.32 [1.28, 1.36]	< 1.0 e-324	1.28 [1.23, 1.32]	< 1.0 e-324
$P_{interaction}$[†]		1.4 e-19		1.7 e-17	
	**mRNFL thickness (microns)**				
All	33,112	0.02 [-0.02, 0.06]	0.31	-0.02 [-0.07, 0.02]	0.25
< 51 y	7,908	0.08 [-0.01, 0.16]	0.07	0.05 [-0.04, 0.14]	0.24
51-57 y	7,326	-0.03 [-0.11, 0.06]	0.52	-0.06 [-0.15, 0.03]	0.19
58-62 y	7,663	0.04 [-0.04, 0.13]	0.28	0.03 [-0.06, 0.11]	0.55
≥ 63 y	10,215	-0.03 [-0.10, 0.04]	0.43	-0.11 [-0.18, -0.03]	0.01
$P_{interaction}$[†]		0.05		8.0 e-03	
	**mGCIPL thickness (microns)**				
All	33,027	-0.20 [-0.25, -0.15]	4.1 e-13	-0.14 [-0.20, -0.08]	1.1 e-06
< 51 y	7,884	-0.12 [-0.23, -0.01]	0.03	-0.09 [-0.21, 0.02]	9.8 e-02
51-57 y	7,310	-0.24 [-0.36, -0.13]	3.4 e-05	-0.16 [-0.29, -0.05]	0.006
58-62 y	7,638	-0.21 [-0.32, -0.10]	0.0002	-0.10 [-0.21, 0.02]	0.1
≥ 63 y	10,195	-0.27 [-0.37, -0.17]	3.3 e-08	-0.20 [-0.30, -0.09]	0.0002
$P_{interaction}$[†]		0.02		0.18	
	**Glaucoma risk**				
	**N case/ N total**	**OR [95% CI] per SD of mtGPRS**	**P-value**	**OR [95% CI] per SD of mtGPRS**	**P-value**
All	7,298/ 154,406	2.87 [2.80, 2.94]	< 0.001	2.88 [2.80, 2.96]	< 0.001
< 51 y	542/ 33,886	2.68 [0.46, 2.92]	< 0.001	2.71 [2.47, 2.98]	2.1 e-96
51-57 y	1,081/ 33,336	2.85 [2.68, 3.03]	< 0.001	2.85 [2.66, 3.05]	1.8 e-191
58-62 y	1,867/ 37,044	3.00 [2.85, 3.15]	< 0.001	2.99 [2.83, 3.16]	< 0.001
≥ 63 y	3,808/ 50,140	2.89 [2.79, 3.00]	< 0.001	2.87 [2.75, 2.99]	< 0.001
$P_{interaction}$[†]		0.13		0.13	

Abbreviations: mtGPRS, multitrait glaucoma polygenic risk score; y, years; IOP, intraocular pressure; RNFL, Retinal nerve fiber layer; GCIPL, Ganglion cell inner plexiform layer; OR, odds ratio; CI, confidence interval.

[*] Categorization based on the quartiles of age in the UK Biobank amongst participants with available IOP measurements.

[†] P-value of the interaction test (Wald test) between mtGPRS and continuous age.

The unadjusted model represents raw computes. The adjusted model controls for age, age^2, sex, smoking status (never, past, current), number of cigarettes (only among current smokers), alcohol consumption (never/special occasion only or ever/frequently), physical activity (MET-hours/week), Townsend Deprivation Index, body mass index. (kg/m^2), systolic blood pressure (mmHg), diabetes, cardiovascular disease, spherical equivalents (diopters), coffee and tea intake, beta-blocker use, and age*deprivation.

S7 Table. Age-stratified associations between trait-specific polygenic risk score (PRS) per standard deviation (SD) and four outcomes among genetically Europeans in the UK Biobank.

	Unadjusted model			Adjusted model	
	IOP (mm Hg)				
**Age***	**N**	**Beta [95% CI] per SD of IOP PRS**	**P-value**	**Beta [95% CI] per SD of IOP PRS**	**P-value**
All	92,693	0.74 [0.72, 0.76]	< 1.0 e-324	0.72 [0.70, 0.75]	< 1.0 e-324
< 51 y	20,300	0.66 [0.61, 0.71]	1.5 e-164	0.66 [0.61, 0.71]	3.4 e-148
51-57 y	21,197	0.67 [0.62, 0.71]	6.9 e-153	0.63 [0.58, 0.68]	5.4 e-120
58-62 y	22,549	0.76 [0.71, 0.81]	9.3 e-199	0.74 [0.69, 0.79]	4.1 e-162
≥ 63 y	29,647	0.82 [0.78, 0.87]	7.3 e-287	0.82 [0.77, 0.86]	6.3 e-234
$P_{interaction}$†		7.4 e-09		5.1 e-08	
	mRNFL thickness (microns)				
	**N**	**Beta [95% CI] per SD of mRNFL PRS**	**P-value**	**Beta [95% CI] per SD of mRNFL PRS**	**P-value**
All	33,112	0.73 [0.69, 0.77]	3.2 e-269	0.73 [0.69, 0.77]	1.2 e-233
< 51 y	7,908	0.78 [0.70, 0.86]	5.5 e-76	0.79 [0.70, 0.87]	2.4 e-68
51-57 y	7,326	0.72 [0.63, 0.80]	2.5 e-59	0.73 [0.63, 0.82]	3.3 e-52
58-62 y	7,663	0.76 [0.67, 0.84]	3.4 e-69	0.76 [0.67, 0.85]	1.7 e-61
≥ 63 y	10,215	0.68 [0.61, 0.76]	1.9 e-74	0.66 [0.58, 0.74]	5.4 e-58
$P_{interaction}$†		0.09		0.02	
	mGCIPL thickness (microns)				
	**N**	**Beta [95% CI] per SD of mGCIPL PRS**	**P-value**	**Beta [95% CI] per SD of mGCIPL PRS**	**P-value**
All	33,027	1.14 [1.09, 1.20]	< 1.0 e-324	1.16 [1.11, 1.22]	< 1.0 e-324
< 51 y	7,884	1.19 [1.08, 1.30]	2.9 e-103	1.18 [1.07, 1.29]	1.4 e-94
51-57 y	7,310	1.22 [1.10, 1.34]	7.5 e-91	1.29 [1.17, 1.42]	7.8 e-95
58-62 y	7,638	1.11 [0.99, 1.23]	1.8 e-77	1.09 [0.97, 1.22]	4.5 e-68
≥ 63 y	10,195	1.11 [1.01, 1.21]	3.6 e-105	1.10 [1.00, 1.21]	4.9 e-90
$P_{interaction}$†		0.05		0.06	
	Glaucoma risk				
	**N case/ N total**	**OR [95% CI] per SD of glaucoma PRS**	**P-value**	**OR [95% CI] per SD of glaucoma PRS**	**P-value**
All	7,298/ 154,406	1.69 [1.65, 1.73]	< 0.001	1.71 [1.67, 1.76]	< 0.001
< 51 y	542/ 33,886	1.61 [1.48, 1.75]	< 0.001	1.58 [1.44, 1.73]	1.8 e-21
51-57 y	1,081/ 33,336	1.72 [1.62, 1.83]	< 0.001	1.71 [1.59, 1.83]	2.0 e-52
58-62 y	1,867/ 37,044	1.71 [1.63, 1.79]	< 0.001	1.73 [1.64, 1.83]	2.9 e-91
≥ 63 y	3,808/ 50,140	1.71 [1.65, 1.76]	< 0.001	1.73 [1.67, 1.80]	9.3 e-166
$P_{interaction}$†		0.35		0.06	

Abbreviations: IOP, intraocular pressure; y, years; mRNFL, macula region retinal nerve fiber layer; mGCIPL, macula region ganglion cell inner plexiform layer; OR, odds ratio; CI, confidence interval.

* Categorization based on the quartiles of age in the full UK Biobank amongst participants with available IOP measurements.

† P-value of the interaction test (Wald test) between trait-specific GRS and continuous age.

The unadjusted model represents raw computes. The adjusted model controls for age, age^2, sex, smoking status (never, past, current), number of cigarettes (only among current smokers), alcohol consumption (never/special occasion only or ever/frequently), physical activity (MET-hours/week), Townsend Deprivation Index, body mass index. (kg/m^2), systolic blood pressure (mmHg), diabetes, cardiovascular disease, spherical equivalents (diopters), coffee and tea intake, beta-blocker use, and age*deprivation. See the supplemental SNP lists for gene variants comprising the trait-specific PRSs.

S8 Table. Sensitivity analysis for the association between the mtGPRS per standard deviation (SD) of continuous glaucoma traits (IOP, mRNFL, and mGCIPL) after excluding participants with glaucoma at baseline.

Age*	All UKB participants			Genetically Caucasian only		
	IOP (mm Hg)					
	N	Beta [95% CI] per SD of mtGPRS	$P_{interaction}$†	N	Beta [95% CI] per SD of mtGPRS	$P_{interaction}$†
All	116,287	1.02 [1.00, 1.04]	4.9 e-12	91,286	1.07 [1.05, 1.09]	9.3 e-07
< 51 years	29,210	0.92 [0.88, 0.96]		20,200	0.98 [0.94, 1.03]	
51-57 years	25,996	0.97 [0.92, 1.01]		20,010	1.02 [0.97, 1.07]	
58-62 years	26,923	1.08 [1.03, 1.12]		22,197	1.12 [1.07, 1.17]	
≥ 63 years	34,158	1.10 [1.06, 1.14]		28,879	1.13 [1.09, 1.17]	
	**mRNFL thickness (microns)**					
All	41,601	-0.01 [-05, 0.03]	0.05	32,703	-0.004 [-0.05, 0.04]	0.04
< 51 years	11,300	0.05 [-0.02, 0.13]		7,878	0.06 [-0.03, 0.15]	
51-57 years	9,428	-0.06 [-0.14, 0.02]		7,267	-0.04 [-0.13, 0.05]	
58-62 years	9,220	0.02 [-0.06, 0.10]		7,567	0.03 [-0.06, 0.12]	
≥ 63 years	11,653	-0.05 [-0.12, 0.03]		9,991	-0.06 [-0.14, 0.02]	
	**mGCIPL thickness (microns)**					
All	41,511	-0.11 [-0.16, -0.06]	0.64	32,618	-0.11 [-0.17, -0.05]	0.58
< 51 years	11,261	-0.08 [-0.18, 0.01]		7,853	-0.09 [-0.21, 0.02]	
51-57 years	9,406	-0.16 [-0.27, -0.05]		7,250	-0.15 [-0.27, -0.03]	
58-62 years	9,196	-0.09 [-0.20, 0.02]		7,542	-0.07 [-0.19, 0.05]	
≥ 63 years	11,648	-0.13 [-0.23, -0.03]		9,973	-0.14 [-0.24, -0.03]	

Abbreviations: mtGPRS, multitrait glaucoma polygenic risk score; IOP, intraocular pressure; mRNFL, macula region retinal nerve fiber layer; mGCIPL, macula region ganglion cell inner plexiform layer.

* Categorization based on the quartiles of age in the full UK biobank amongst participants with available IOP measurements.

† P-value of the interaction test (Wald test) between mtGPRS and continuous age.

Adjusted for age, age^2, sex, principal component analysis-based ancestry (it was excluded in the analysis for genetically Caucasian only), smoking status (never, past, current) number of cigarettes (only among current smokers), alcohol consumption (never/special occasion only or ever/frequently), physical activity (MET-hours/week), Townsend Deprivation Index, body mass index (kg/m^2), systolic blood pressure (mmHg), diabetes mellitus, cardiovascular disease, spherical equivalents (diopters), coffee and tea intake, beta-blocker use, and age*deprivation.

S9 Table. Sensitivity analysis for the association between the mtGPRS per Standard Deviation (SD) and glaucoma traits (IOP, mRNFL, mGCIPL and glaucoma) after excluding participants with diabetes mellitus.

Age*	Full UKB participants			Genetically European only		
	IOP (mm Hg)					
	N	Beta [95% CI] per SD of mtGPRS	$P_{interaction}$†	N	Beta [95% CI] per SD of mtGPRS	$P_{interaction}$†
All	111,083	1.10 [1.08, 1.13]	4.5 e-28	87,739	1.16 [1.14, 1.19]	8.9 e-18
< 51 y	28,360	0.94 [0.90, 0.98]		19,753	1.02 [0.97, 1.06]	
51-57 y	24,868	1.02 [0.97, 1.06]		19,276	1.08 [1.03, 1.13]	
58-62 y	25,614	1.18 [1.14, 1.23]		21,251	1.23 [1.18, 1.28]	
≥ 63 y	32,241	1.24 [1.20, 1.29]		27,459	1.28 [1.23, 1.13]	
	mRNFL (microns)					
All	39,958	-0.03 [-0.07, 0.01]	0.01	31,552	-0.03 [-0.07, 0.01]	0.004
< 51 y	11,003	0.05 [-0.03, 0.12]		7,724	0.05 [-0.04, 0.14]	
51-57 y	9,079	-0.08 [-0.16, 0.00]		7,030	-0.06 [-0.15, 0.04]	
58-62 y	8,786	0.01 [-0.08, 0.09]		7,252	0.02 [-0.07, 0.11]	
≥ 63 y	11,090	-0.10 [-0.17, -0.02]		9,546	-0.13 [-0.21, -0.04]	
	mGCIPL (microns)					
All	39,872	-0.13 [-0.18, -0.08]	0.28	31,468	-0.13 [-0.19, -0.08]	0.31
< 51 y	10,970	-0.08 [-0.18, 0.02]		7,702	-0.09 [-0.20, 0.02]	
51-57 y	9,054	-0.18 [-0.29, -0.07]		7,013	-0.17 [-0.30, -0.05]	
58-62 y	8,766	-0.10 [-0.21, 0.01]		7,231	-0.09 [-0.21, 0.02]	
≥ 63 y	11,082	-0.16 [-0.26, -0.06]		9,522	-0.17 [-0.28, -0.07]	
	Glaucoma risk					
	N	OR [95% CI] per SD of mtGPRS	$P_{interaction}$†	N	OR [95% CI] per SD of mtGPRS	$P_{interaction}$†
All	179,232	2.75 [2.68, 2.83]	0.003	144,784	2.92 [2.84, 3.01]	0.27
< 51 y	45,079	2.49 [2.29, 2.71]		32,776	2.76 [2.51, 3.05]	
51-57 y	39,825	2.66 [2.49, 2.84]		31,506	2.91 [2.71, 3.13]	
58-62 y	41,004	2.85 [2.70, 3.01]		34,587	3.04 [2.86, 3.22]	
≥ 63 y	53,324	2.80 [2.69, 2.91]		45,915	2.90 [2.78, 3.03]	

Abbreviations: mtGPRS, multitrait glaucoma polygenic risk score; y, years; IOP, intraocular pressure; RNFL, Retinal nerve fiber layer; GCIPL, Ganglion cell inner plexiform layer.

* Categorization based on the quartiles of age in the full UK biobank participants with IOP measurements.

† P-value of the interaction test (Wald test) between multitrait GPRS and continuous age

Adjusted for age, age^2, sex, principal component analysis-based ancestry, smoking status (never, past, current) number of cigarettes (only among current smokers), alcohol consumption (never/special occasion only or ever/frequently), physical activity (MET-hours/week), Townsend Deprivation Index, body mass index (kg/m^2), systolic blood pressure (mmHg), cardiovascular disease, spherical equivalents (diopters), coffee and tea intake, beta-blocker use, and age*deprivation.

S10 Table. Age-stratified associations between IOP- and glaucoma-related SNPs and IOP and glaucoma risk with SNP-age interactions in UK Biobank participants.[*]

	< 51 years	51-57 years	58-62 years	≥ 63 years	
	**IOP (mmHg)**				
**IOP-related gene**	**Beta [95% CI]**	**Beta [95% CI]**	**Beta [95% CI]**	**Beta [95% CI]**	$P_{interaction}$[†]
**(N)**	29,354	26,271	27,392	35,136	
MYOC (Myocilin)					
rs74315329[‡]	1.64 [0.64, 2.64]	1.49 [0.51, 2.47]	1.96 [0.94, 2.98]	2.67 [1.75, 3.60]	0.05
TMCO1					
rs116089225[§]	0.77 [0.43, 1.10]	0.47 [0.10, 0.84]	0.60 [0.24, 0.97]	0.89 [0.55, 1.25]	0.38
rs10918274[‖]	0.34 [0.25, 0.44]	0.33 [0.23, 0.44]	0.47 [0.36, 0.58]	0.46 [0.36, 0.56]	0.01
	**Glaucoma risk**				
**Glaucoma-related gene**	**OR [95% CI]**	**OR [95% CI]**	**OR [95% CI]**	**OR [95% CI]**	$P_{interaction}$[†]
**(#cases/controls)**	784/46,119	1,391/41,048	2,254/41,963	4,553/54,171	
CDKN2B-AS1					
rs10965235[#]	1.10 [0.82, 1.49]	1.07 [0.80, 1.42]	0.75 [0.54, 1.04]	1.31 [1.05, 1.63]	0.98
SIX6					
rs33912345[**]	1.08 [0.96, 1.21]	1.13 [1.08, 1.20]	1.12 [1.05, 1.20]	1.01 [1.01, 1.01]	0.39
**IOP-related gene**					
MYOC (Myocilin)					
rs74315329[‡]	2.53 [0.79, 8.05]	4.81 [3.20, 7.24]	5.15 [3.07, 8.65]	1.27 [1.21, 1.33]	0.09
TMCO1					
rs116089225[§]	2.60 [1.72, 3.92]	1.47 [1.16, 1.87]	1.43 [1.06, 1.94]	1.04 [1.02, 1.06]	0.13
rs10918274[‖]	1.36 [1.17, 1.59]	1.35 [1.25, 1.45]	1.32 [1.20, 1.45]	1.02 [1.02, 1.03]	0.70

Abbreviations: SNP, single nucleotide polymorphism; IOP, intraocular pressure; y, years; CI, confidence interval; ca=cases; co=controls.

Interpretation: mean difference in IOP (95% CI) or odds ratio of glaucoma risk (95% CI) per effect allele of the SNP.

[*] Categorization based on the quartiles of age in the full UK Biobank amongst participants with available IOP measurements.

[†] P-value of the interaction test (Wald test) between each SNP and continuous age.

[‡] Effect allele of the rs74315329 was A (mean allele frequency = 0.003 among UKB participants with IOP; same frequency in UK Biobank participants with glaucoma data).

[§] Effect allele of the rs116089225 (in IOP GRS) was T (mean allele frequency = 0.02 among UKB participants with IOP; same frequency in UK Biobank participants with glaucoma data).

[‖] Effect allele of the rs10918274 (in IOP GRS) was T (mean allele frequency = 0.24 among UKB participants with IOP; same frequency in UK Biobank participants with glaucoma data).

[#] Effect allele of the rs10965235 was C (mean allele frequency = 0.04 among UK Biobank participants with glaucoma data).

[**] Effect allele of the rs33912345 was C (mean allele frequency = 0.84 among UK Biobank participants with glaucoma data).

Adjusted for age, age^2, sex, principal component analysis-based ancestry, smoking status (never, past, current), number of cigarettes (only among current smokers), alcohol consumption (never/special occasion only or ever/frequently), physical activity (MET-hours/week), Townsend Deprivation Index, body mass index.

(kg/m^2), systolic blood pressure (mmHg), diabetes, cardiovascular disease, spherical equivalents (diopters), coffee and tea intake, beta-blocker use, and age*deprivation.

S11 Table. Age-stratified associations between trait-specific polygenic risk scores (PRSs) and four outcomes by sex among UK Biobank participants.

Age*	Male N	Male Beta [95% CI] per SD of IOP PRS	Male $P_{interaction}$†	Female N	Female Beta [95% CI] per SD of IOP PRS	Female $P_{interaction}$†	$P_{interaction}$‡
	**IOP (mm Hg)**						
All	54,974	0.72 [0.69, 0.76]	8.9 e-06	63,179	0.66 [0.63, 0.69]	1.4 e-08	0.43
< 51 years	13,305	0.65 [0.58, 0.71]		16,049	0.58 [0.53, 0.64]		
51-57 years	11,484	0.66 [0.59, 0.73]		14,787	0.57 [0.51, 0.63]		
58-62 years	12,572	0.74 [0.67, 0.81]		14,820	0.67 [0.61, 0.74]		
≥ 63 years	17,613	0.81 [0.75, 0.87]		17,523	0.81 [0.75, 0.87]		

Age*	Male N	Male Beta [95% CI] per SD of RNFL PRS	Male $P_{interaction}$†	Female N	Female Beta [95% CI] per SD of RNFL PRS	Female $P_{interaction}$†	$P_{interaction}$‡
	**mRNFL thickness (microns)**						
All	19,939	0.69 [0.64, 0.75]	0.31	22,193	0.70 [0.65, 0.75]	0.59	0.71
< 51 years	5,344	0.75 [0.64, 0.85]		5,996	0.68 [0.57, 0.78]		
51-57 years	4,237	0.68 [0.56, 0.80]		5,270	0.71 [0.60, 0.82]		
58-62 years	4,314	0.67 [0.54, 0.79]		5,030	0.81 [0.70, 0.91]		
≥ 63 years	6,044	0.66 [0.56, 0.77]		5,897	0.63 [0.52, 0.74]		

Age*	Male N	Male Beta [95% CI] per SD of GCIPL PRS	Male $P_{interaction}$†	Female N	Female Beta [95% CI] per SD of GCIPL PRS	Female $P_{interaction}$†	$P_{interaction}$‡
	**mGCIPL thickness (microns)**						
All	19,868	1.12 [1.04, 1.19]	0.09	22,174	1.15 [1.08, 1.22]	0.87	0.22
< 51 years	5,301	1.13 [0.99, 1.27]		6,001	1.06 [0.93, 1.19]		
51-57 years	4,220	1.24 [1.07, 1.40]		5,265	1.27 [1.12, 1.41]		
58-62 years	4,308	1.10 [0.94, 1.27]		5,012	1.12 [0.97, 1.28]		
≥ 63 years	6,039	1.03 [0.89, 1.17]		5,896	1.15 [1.02, 1.29]		

Age*	Male N case/ N total	Male OR [95% CI] per SD of glaucoma PRS	Male $P_{interaction}$†	Female N case/ N total	Female OR [95% CI] per SD of glaucoma PRS	Female $P_{interaction}$†	$P_{interaction}$‡
	**Glaucoma risk**						
All	4,680/ 88,713	1.67 [1.62, 1.73]	0.049	4,302/ 103,570	1.65 [1.60, 1.72]	0.002	0.25
< 51 years	422/ 21,483	1.56 [1.40, 1.73]		362/ 25,420	1.41 [1.25, 1.59]		
51-57 years	706/ 18,668	1.68 [1.54, 1.83]		685/ 23,771	1.57 [1.44, 1.72]		
58-62 years	1,147/ 19,995	1.67 [1.56, 1.79]		1,107/ 24,222	1.70 [1.59, 1.82]		
≥ 63 years	2,405/ 28,567	1.70 [1.62, 1.78]		2,148/ 30,157	1.72 [1.63, 1.82]		

Abbreviations: GRS, genetic risk score; IOP, intraocular pressure; mRNFL, macula region retinal nerve fiber layer; mGCIPL, macula region ganglion cell inner plexiform layer; OR, odds ratio; CI, confidence interval.

* Categorization based on the quartiles of age in the full UK biobank amongst participants with available IOP measurements.

† P-value of the interaction test (Wald test) between trait-specific GRS and continuous age.
‡ P-value of the three-way interaction test (Wald test) of trait-specific PRS, continuous age, and sex.
Adjusted for age, age^2, principal component analysis-based ancestry, smoking status (never, past, current), number of cigarettes (only among current smokers), alcohol consumption (never/special occasion only or ever/frequently), physical activity (MET-hours/week), Townsend Deprivation Index, body mass index. (kg/m^2), systolic blood pressure (mmHg), diabetes, cardiovascular disease, spherical equivalents (diopters), coffee and tea intake, beta-blocker use, and age*deprivation

S12 Table. Age-stratified associations between multitrait GPRS and four outcomes by principal component analysis-based ancestry groups among UK Biobank participants.

	UK			Other European			Asian		
	IOP (mm Hg)								
Age*	N	beta [95% CI]	$P_{interaction}$†	N	beta [95% CI]	$P_{interaction}$†	N	beta [95% CI]	$P_{interaction}$†
All	102,759	1.15 [1.13, 1.17]	6.4 e-21	3,898	0.82 [0.69, 0.95]	0.49	3,321	0.70 [0.54, 0.86]	0.29
< 51 y	23,189	1.00 [0.96, 1.04]		1,225	0.80 [0.58, 1.02]		1,319	0.78 [0.55, 1.01]	
51-57 y	22,500	1.06 [1.01, 1.11]		933	0.97 [0.73, 1.21]		843	0.78 [0.45, 1.12]	
58-62 y	24,738	1.22 [1.17, 1.26]		871	0.53 [0.26, 0.82]		563	0.41 [-0.02, 0.83]	
≥ 63 y	32,332	1.27 [1.23, 1.31]		869	1.03 [0.70, 1.35]		596	0.69 [0.28, 1.09]	
	mRNFL thickness (microns)								
All	36,810	-0.03 [-0.07, 0.01]	0.02	1,448	0.05 [-0.18, 0.28]	0.47	939	-0.20 [-0.49, 0.10]	0.2
< 51 y	9,076	0.04 [-0.04, 0.12]		491	0.24 [-0.13, 0.62]		404	-0.26 [-0.68, 0.17]	
51-57 y	8,215	-0.06 [-0.14, 0.03]		331	-0.25 [-0.73, 0.23]		237	-0.58 [-1.27, 0.14]	
58-62 y	8,427	0.02 [-0.06, 0.10]		328	-0.02 [-0.52, 0.48]		165	0.07 [-0.73, 0.86]	
≥ 63 y	11,092	-0.10 [-0.17, -0.02]		298	0.07 [-0.48, 0.63]		133	0.31 [-0.59, 1.20]	
	mGCIPL thickness (microns)								
All	36,725	-0.13 [-0.18, -0.08]	0.35	1,451	-0.19 [-0.48, 0.11]	0.06	959	-0.15 [-0.55, 0.25]	0.26
< 51 y	9,051	-0.10 [-0.20, 0.01]		492	0.13 [-0.36, 0.62]		399	-0.32 [-0.92, 0.29]	
51-57 y	8,192	-0.16 [-0.27, -0.05]		332	-0.05 [-0.68, 0.57]		235	-0.11 [-1.09, 0.85]	
58-62 y	8,405	-0.09 [-0.20, 0.02]		328	-0.48 [-1.10, 0.15]		165	-0.29 [-1.18, 0.60]	
≥ 63 y	11,077	-0.17 [-0.27, -0.07]		299	-0.71 [-1.40, -0.01]		136	0.77 [-0.46, 2.00]	
	Glaucoma risk								
	N case / N total	OR [95% CI]		N case / N total	OR [95% CI]		N case / N total	OR [95% CI]	
All	7,973/170,163	2.84 [2.77, 2.92]	0.92	220/ 6,057	1.67 [1.43, 1.96]	0.63	199/ 4,674	1.63 [1.34, 1.98]	0.63
< 51 y	621/ 38,305	2.69 [2.47, 2.94]		22/ 1,806	1.36 [0.81, 2.25]		29/ 1,836	1.80 [1.08, 3.01]	
51-57 y	1,186/ 37,008	2.81 [2.63, 3.00]		33/ 1,483	1.77 [1.22, 2.59]		49/ 1,195	0.90 [0.59, 1.38]	
58-62 y	2,030/ 40,375	2.91 [2.76, 3.06]		60/ 1,368	1.89 [1.37, 2.59]		44/ 776	1.82 [1.16, 2.86]	
≥ 63 y	4,136/ 54,475	2.86 [2.75, 2.97]		105/ 1,400	1.62 [1.26, 2.08]		77/ 867	2.21 [1.58, 3.08]	

Abbreviations: mtGPRS, multitrait analysis of GWAS polygenic risk score; y, years; IOP, intraocular pressure; mRNFL, macula region retinal nerve fiber layer; mGCIPL, macula region ganglion cell inner plexiform layer; OR, odds ratio; CI, confidence interval.

* Categorization based on the quartiles of age in the full UK biobank amongst participants with available IOP measurements.

† P-value of the interaction test (Wald test) between mtGPRS and continuous age.

‡ P-value of the three-way interaction (Wald test) of mtGPRS, continuous age, and sex.

Adjusted for age, age^2, sex, smoking status, number of cigarettes (only among current smokers), alcohol consumption (never/special occasion only or ever/frequently), physical activity (MET-hours/week), Townsend Deprivation Index, body mass index. (kg/m^2), systolic blood pressure (mmHg), diabetes, cardiovascular disease, spherical equivalents (diopters), coffee and tea intake, beta-blocker use, and age*deprivation; Ancestral groups were determined as described in the methods.

S12 Table. Age-stratified associations between multitrait GPRS and four outcomes by principal component analysis-based ancestry groups among UK Biobank participants (continued).

	**African**			**Others**			
	**IOP (mm Hg)**						
Age[a]	N	beta [95% CI]	$P_{interaction}$†	N	beta [95% CI]	$P_{interaction}$†	$P_{interaction}$‡
All	1,885	0.50 [0.22, 0.78]	0.46	6,290	0.59 [0.47, 0.70]	0.17	0.09
< 51 y	908	0.65 [0.29, 1.00]		2,713	0.51 [0.34, 0.67]		
51-57 y	487	-0.40 [-0.99, 0.19]		1,508	0.52 [0.28, 0.76]		
58-62 y	226	1.58 [0.61, 2.56]		994	0.82 [0.52, 1.14]		
≥ 63 y	264	0.52 [-0.41, 1.45]		1,075	0.61 [0.32, 0.90]		
	**mRNFL thickness (microns)**						
All	687	0.31 [-0.09, 0.71]	0.63	2,248	0.56 [0.37, 0.75]	0.08	0.43
< 51 y	339	0.28 [-0.29, 0.85]		1,030	0.46 [0.18, 0.73]		
51-57 y	199	0.09 [-0.73, 0.91]		525	0.51 [0.10, 0.92]		
58-62 y	74	0.41 [-0.88, 1.71]		350	0.64 [0.16, 1.12]		
≥ 63 y	75	0.49 [-1.26, 2.25]		343	1.01 [0.52, 1.50]		
	**mGCIPL thickness (microns)**						
All	689	-0.06 [-0.65, 0.53]	0.92	2,242	0.54 [0.35, 0.73]	0.10	0.32
< 51 y	339	-0.40 [-1.21, 0.41]		1,021	0.47 [0.19, 0.74]		
51-57 y	201	0.65 [-0.60, 1.90]		525	0.43 [0.01, 0.85]		
58-62 y	72	-0.38 [-2.74, 1.98]		350	0.65 [0.17, 1.13]		
≥ 63 y	77	-0.70 [-3.26, 1.86]		346	0.97 [0.47, 1.46]		
	**Glaucoma risk**						
	N case / N total	OR [95% CI]		N case / N total	OR [95% CI]		
All	184/ 2,632	1.25 [0.97, 1.60]	0.54	406/ 8,757	1.36 [1.19, 1.57]	0.04	0.38
< 51 y	40/ 1,232	0.95 [0.57, 1.58]		72/ 3,724	1.07 [0.79, 1.46]		
51-57 y	42/ 668	1.31 [0.77, 2.30]		81/ 2,085	1.21 [0.90, 1.64]		
58-62 y	33/ 309	1.68 [0.84, 3.33]		87/ 1,389	1.82 [1.35, 2.46]		
≥ 63 y	69/ 423	1.35 [0.85, 2.15]		166/ 1,559	1.45 [1.15, 1.84]		

Abbreviations: mtGPRS, multitrait analysis of GWAS polygenic risk score; y, years; IOP, intraocular pressure; mRNFL, macula region retinal nerve fiber layer; mGCIPL, macula region ganglion cell inner plexiform layer; OR, odds ratio; CI, confidence interval.

[*] Categorization based on the quartiles of age in the full UK biobank amongst participants with available IOP measurements.

† P-value of the interaction test (Wald test) between mtGPRS and continuous age.

‡ P-value of the three-way interaction (Wald test) of mtGPRS, continuous age, and sex.

Adjusted for age, age^2, sex, smoking status, number of cigarettes (only among current smokers), alcohol consumption (never/special occasion only or ever/frequently), physical activity (MET-hours/week), Townsend Deprivation Index, body mass index. (kg/m^2), systolic blood pressure (mmHg), diabetes, cardiovascular disease, spherical equivalents (diopters), coffee and tea intake, beta-blocker use, and age*deprivation; Ancestral groups were determined as described in the methods.

S13 Table. Genetic heritability of IOP by age quartiles among European UK Biobank participants after excluding any related individuals.

(Age*)	SNP heritability (h^2)
**< 51 y**	0.17
**51-58 y**	0.13
**59-63 y**	0.14
**≥ 64 y**	0.16

* Categorization based on the quartiles of age in the European participants with IOP excluding any related individuals.

Adjusted for age, age^2, sex, smoking status, number of cigarettes (only among current smokers), alcohol, physical activity, deprivation, BMI, SBP, diabetes, CVD, SE, coffee and tea intake, beta-blocker use, age*deprivation, and an indicator variable for UK participants, and top 10 PCs.

S14 Table. Age-stratified prevalence of glaucoma as a function of genetically inferred ancestry groups among UK Biobank participants (N=183,526; 2006-2010).

Age (Years)	African	Asian	UK European	Other European
<51	3.20%	1.60%	1.60%	1.20%
51-57	6.30%	4.10%	3.20%	2.20%
58-62	10.70%	5.70%	5.00%	4.40%
63+	16.30%	8.90%	7.60%	7.50%
Overall prevalence	184/2,632 = 7%	199/4,674 = 4.30%	7,943/170,163 = 4.70%	220/6,057 = 3.60%

Glaucoma and genetically inferred ancestry are determined as outlined in the methods. These numbers are derived from Supplementary Table 12.
